# c‐Myc–IMPDH1/2 axis promotes tumourigenesis by regulating GTP metabolic reprogramming

**DOI:** 10.1002/ctm2.1164

**Published:** 2023-01-11

**Authors:** Qiang Zhang, Kaisa Cui, Xiaoya Yang, Qilang He, Jing Yu, Li Yang, Gang Yao, Weiwei Guo, Zhanhao Luo, Yugeng Liu, Yuan Chen, Zhen He, Ping Lan

**Affiliations:** ^1^ The Sixth Affiliated Hospital School of Medicine Sun Yat‐sen University Guangzhou Guangdong China; ^2^ Wuxi Cancer Institute Affiliated Hospital of Jiangnan University Wuxi Jiangsu China; ^3^ Guangdong Provincial Key Laboratory of Colorectal and Pelvic Floor Diseases, Guangdong Institute of Gastroenterology Guangzhou Guangdong China; ^4^ Zhumadian Central Hospital Huanghuai University Zhumadian Henan China; ^5^ The People's Hospital of Zhengyang County Zhumadian Henan China; ^6^ Center for Synthetic Microbiome Institute of Synthetic Biology Shenzhen Institutes of Advanced Technology Chinese Academy of Sciences Shenzhen Guangdong China

**Keywords:** c‐Myc, colorectal carcinoma, GTP, IMPDH1, IMPDH2, metabolic rate‐limiting enzymes

## Abstract

**Background:**

Metabolic reprogramming is a hallmark of cancer. Metabolic rate‐limiting enzymes and oncogenic c‐Myc (Myc) play critical roles in metabolic reprogramming to affect tumourigenesis. However, a systematic assessment of metabolic rate‐limiting enzymes and their relationship with Myc in human cancers is lacking.

**Methods:**

Multiple Pan‐cancer datasets were used to develop the transcriptome, genomic alterations, clinical outcomes and Myc correlation landscapes of 168 metabolic rate‐limiting enzymes across 20 cancers. Real‐time quantitative PCR and immunoblotting were, respectively, used to examine the mRNA and protein of inosine monophosphate dehydrogenase 1 (IMPDH1) in human colorectal cancer (CRC), azoxymethane/dextran sulphate sodium‐induced mouse CRC and spontaneous intestinal tumours from APC^Min/+^ mice. Clone formation, CCK‐8 and subcutaneous xenograft model were applied to investigate the possible mechanisms connecting IMPDH1 to CRC growth. Co‐immunoprecipitation and protein half‐life assay were used to explore the mechanisms underlying the regulation of IMPDH1.

**Results:**

We explored the global expression patterns, dysregulation profiles, genomic alterations and clinical relevance of 168 metabolic rate‐limiting enzymes across human cancers. Importantly, a series of enzymes were associated with Myc, especially top three upregulated enzymes (TK1, RRM2 and IMPDH1) were positively correlated with Myc in multiple cancers. As a proof‐of‐concept exemplification, we demonstrated that IMPDH1, a rate‐limiting enzyme in GTP biosynthesis, is highly upregulated in CRC and promotes CRC growth in vitro and in vivo. Mechanistically, IMPDH2 stabilizes IMPDH1 by decreasing the polyubiquitination levels of IMPDH1, and Myc promotes the de novo GTP biosynthesis by the transcriptional activation of IMPDH1/2. Finally, we confirmed that the Myc–IMPDH1/2 axis is dysregulated across human cancers.

**Conclusions:**

Our study highlights the essential roles of metabolic rate‐limiting enzymes in tumourigenesis and their crosstalk with Myc, and the Myc–IMPDH1/2 axis promotes tumourigenesis by altering GTP metabolic reprogramming. Our results propose the inhibition of IMPDH1 as a viable option for cancer treatment.

## Graphical abstract

- Global expression patterns, dysregulation profiles, genomic alterations and clinical relevance of 168 metabolic rate‐limiting enzymes and their relationship with Myc were determined in human cancers.
- Metabolic rate‐limiting enzyme IMPDH2 interacts with IMPDH1 to stabilize IMPDH1.
- Myc promotes de novo GTP biosynthesis in human cancers by transcriptional activation of IMPDH1 and IMPDH2.

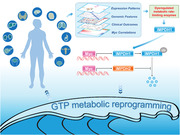

## 1 INTRODUCTION

Metabolic reprogramming is critical for malignant transformation and tumour progression, including tumour growth, metastasis, angiogenesis and drug resistance.^1^, ^2^ Metabolic rate‐limiting enzymes not only affect the speed of the metabolic pathway but can also change the metabolic direction, implying their critical roles in metabolic reprogramming. Various studies have indicated that the dysregulation of metabolic rate‐limiting enzymes affects tumour development and progression by altering metabolic reprogramming.^3^, ^4^, ^5^, ^6^, ^7^ Therefore, identification of the dysregulated metabolic rate‐limiting enzymes in human cancers may provide useful prognostic biomarkers and therapeutic targets.

c‐Myc (Myc) is a frequently activated oncogene in human cancers. Oncogenic Myc has appeared to share metabolic targets involved in various metabolic processes, such as glycolysis, glutaminolysis, lipid synthesis and de novo nucleotide synthesis.^8^, ^9^, ^10^, ^11^, ^12^ In return, some metabolic enzymes affect tumour initiation and progression by altering Myc signatures.^13^, ^14^ However, the regulatory relationship between metabolic rate‐limiting enzymes and Myc remains unclear.

Inosine monophosphate dehydrogenase 1/2 (IMPDH1/2), a rate‐limiting enzyme in GTP biosynthesis, catalyses a crucial step of converting IMP into XMP that is further converted into GTP. Previous studies have shown that cancer cells rely on GTP to promote rRNA and tRNA syntheses and cell cycle progression.^15^, ^16^, ^17^ In addition, accumulated evidence has revealed the aberrantly high expression of IMPDH2 in many cancers, such as ovarian cancer,^18^ non‐small cell lung cancer,^19^ triple‐negative breast cancer,^20^ glioblastoma,^15^ kidney cancer,^21^ bladder cancer^21^ and colorectal cancer (CRC),^22^ implying that IMPDH2 is a potential therapeutic target for cancer. However, the role of IMPDH1 in cancer, especially in CRC, has been largely ignored because IMPDH1 is less expressed than IMPDH2 in most tissues.^15^, ^23^

Herein, we investigated the transcriptome landscapes, dysregulation profiles, genomic alterations and clinical outcomes related to 168 metabolic rate‐limiting enzymes in primary solid tumours. We also evaluated the correlations between Myc and transcriptome characteristics of these enzymes across human cancers. As a proof‐of‐concept exemplification, we demonstrated that IMPDH1 is significantly  overexpressed in cancer through Pan‐cancer analyses and cellular/molecular experiments. Importantly, IMPDH2 stabilizes IMPDH1 by decreasing the polyubiquitination levels of IMPDH1, and Myc promotes the de novo GTP biosynthesis by transcriptional activation of IMPDH1/2. Moreover, we confirmed that the Myc–IMPDH1/2 axis is dysregulated in human cancers. Here, we propose the inhibition of IMPDH1 as a viable option for cancer treatment.

## 2 MATERIALS AND METHODS

### 2.1 Pan‐cancer analysis

TCGA Pan‐cancer data of 20 human primary tumour samples were downloaded from the official website (http://cancergenome.nih.gov/) and obtained from the previous resource.^24^ Abbreviations of each cancer used in this study are listed in Table S1. A total of 168 metabolic rate‐limiting enzymes were identified by reviewing the literature (Table S2). Protein expression data were downloaded from https://proteomics.cancer.gov/data‐portal (Table S3). Expression of cancer cell lines was downloaded from DepMap (https://depmap.org/portal/). Dysregulated metabolic rate‐limiting enzymes were defined as previously described.^25^ The expressions of these enzymes in CRC are shown in Table S4. Analyses of copy number variations (CNV) gain/loss, DNA methylation and mutation were performed according our previous study.^25^ Overall survival (OS) analyses were performed as our previously described.^25^, ^26^ For details, please see Supporting Information section.

### 2.2 Cell culture and transfection

Human HCT116, HCT18, DLD1, RKO, LOVO, HCT8, SW480, HT29, HIEC‐6 and HEK293T cells were obtained from the American Type Culture Collection (ATCC). Cells were cultured in DMEM medium (Gibco, NY, USA) supplemented with 10% foetal bovine serum (Gibco, NY, USA) and 1% penicillin–streptomycin (Gibco, CA, USA) at 37°C in a 5% CO_2_ incubator. For transfection, after growing to 70% confluence, cells were transfected using Lipofectamine 3000 (Invitrogen, Carlsbad, CA) or HighGene (ABclonal, Wuhan, China), according to the manufacturer's instructions.

### 2.3 Reagents and plasmids

Human GTP ELISA kits (MM‐60800H2) were purchased from Meimian. GTP powder (ST1362) was purchased from Beyotime. Cycloheximide (CHX, GC17198) was purchased from Glpbio. CCK‐8 kits (BS350B) were purchased from Guangzhou Barley Biotechnology (China). Anti‐Flag agarose beads (23101) were purchased from Selleck (Houston, USA). Anti‐HA agarose beads (KTSM1305) were purchased from Shenzhen KangTi Life Technology (China). All antibody information are identified in Table S5. The human IMPDH1 and IMPDH2 coding sequences were amplified from HEK293T cDNA and cloned into pHAGE‐CMV‐MCS‐PGK and pCMV‐HA vectors, respectively. Deletion mutants of IMPDH1 and IMPDH2 were inserted into the pHAGE‐CMV‐MCS‐PGK and pCMV‐HA vectors, respectively. Human shIMPDH1‐1# and shIMPDH1‐2# were synthesized by RuiBiotech (Guangzhou, China), then annealed and cloned into the pLKO.1‐puro vector. Human shMyc‐1# and shMyc‐2# were purchased from MiaoLingPlasmid. All plasmid primers are presented in Table S6.

### 2.4 Human CRC specimens

A total of 35 fresh CRC samples and matched normal adjacent tissues were used to analyse IMPDH1, MYLK, XDH, DPYD, UGDH and PTGS1 transcript levels. A total of 20 fresh CRC samples and matched normal adjacent tissues were used to analyse IMPDH1, IMPDH2 and Myc; GTP levels were detected in 12 out of 20 clinical samples. All patient samples were obtained from the Sixth Affiliated Hospital of Sun Yat‐sen University.

### 2.5 Subcutaneous xenograft model

Subcutaneous xenograft was performed as described previously.^27^ Briefly, a total of 5 × 10^6^ HT29 cells were digested and suspended in 100 μl PBS. The resuspended cells were then injected subcutaneously into the flanks of nude mice (*n* = 5 per group). The shCtrl and shIMPDH1 HT29 cells were injected into the contralateral flanks of the same nude mouse. Tumour growth was monitored every 3 days after 10 days of tumour inoculation. The tumour volume was calculated as the equation *V* (mm^3^) = *a* × *b* ^2^/2 (*a*, length; *b*, width). Tumours were harvested for GTP detection.

### 2.6 Stable cell lines

Stable cell line construction was performed as described previously.^27^ Briefly, indicated lentiviral vectors were packaged in HEK293T cells. HT29 or RKO cells were infected with indicated lentiviruses in the presence of polybrene. The cells were then treated with 1 μg/ml puromycin for 2 weeks to obtain stable cell lines. The IMPDH1 protein expression in stable clones was validated by immunoblotting.

### 2.7 Real‐time quantitative PCR (qRT‐PCR)

Real‐time quantitative PCR (qRT‐PCR) assays were performed as described previously.^27^ Briefly, total RNA was isolated from cells or tissues and subsequent reverse transcription was performed. qPCR was then performed with SYBR Green Supermix (Bio‐Rad, Hercules, CA) using standard procedures. All primer sequences in this study are listed in Table S6. GAPDH was used as an internal control.

### 2.8 Protein half‐life assay

Protein half‐life assays were performed as described previously.^27^ Briefly, cells transfected with the indicated plasmids were treated with CHX (100 μg/ml) for the indicated times before collection. The cells were then lysed and boiled, and proteins were detected by immunoblot with the indicated antibodies.

### 2.9 CCK‐8 assays

For CCK‐8 assays, cells were seeded into 96‐well plates (10^3^ cells/well) and cultured in DMEM supplemented with 10% FBS. Then CCK‐8 assays were performed, according to the manufacturer's instructions.

### 2.10 Co‐immunoprecipitation (Co‐IP) and immunoblot analyses

Co‐immunoprecipitation (Co‐IP) and immunoblot analysis were performed as described previously.^27^ Briefly, cells were lysed in 1 ml lysis buffer. For immunoprecipitation, the anti‐Flag/HA agarose beads were washed with 1 ml lysis buffer three times, and then .95 ml of cell lysate was added to the indicated group and incubated overnight at 4°C. The next day, the agarose beads were centrifuged and washed three times. The agarose beads were then mixed within a 2× SDS sample buffer. Lysate samples were boiled for 10 min and were analysed by immunoblot with the indicated antibodies.

### 2.11 Chromatin immunoprecipitation (ChIP) assay

Cells were cross‐linked in situ with 1% formaldehyde, quenched with .125 M glycine and lysed in lysis buffer. Total lysates were sonicated to crush chromatin DNA to sizes ranging from 200 to 1000 bp. The supernatant was diluted 10‐fold and precleared with 40 μl agarose beads for 3 h at 4°C. Then the indicated antibody (2 μg) was added to the precleared supernatant. The mixture was rotated overnight at 4°C. The next day, 50 μl agarose beads were added and rotated for 3 h at 4°C. Subsequent de‐cross‐linked DNA was subjected to PCR analysis using specific primers listed in Table S6.

### 2.12 Statistical analysis

Mann–Whitney/*t*‐test was used in two‐group comparison. Specific statistical methods were stated in the corresponding methods section or figure legends. All reported *p*‐values were two‐sided. General analysis and visualization were performed using R 4.1.0 with packages of Uniform Manifold Approximation and Projection (UMAP) (0.2.7.0), pheatmap (1.0.12), maftools (2.8.0), survival, and GSVA (1.40.1). Analysis and graphics were performed using R 4.1.0 and GraphPad Prism 9.

## 3 RESULTS

### 3.1 The transcriptome landscape of metabolic rate‐limiting enzymes across human cancers

**FIGURE 1:**
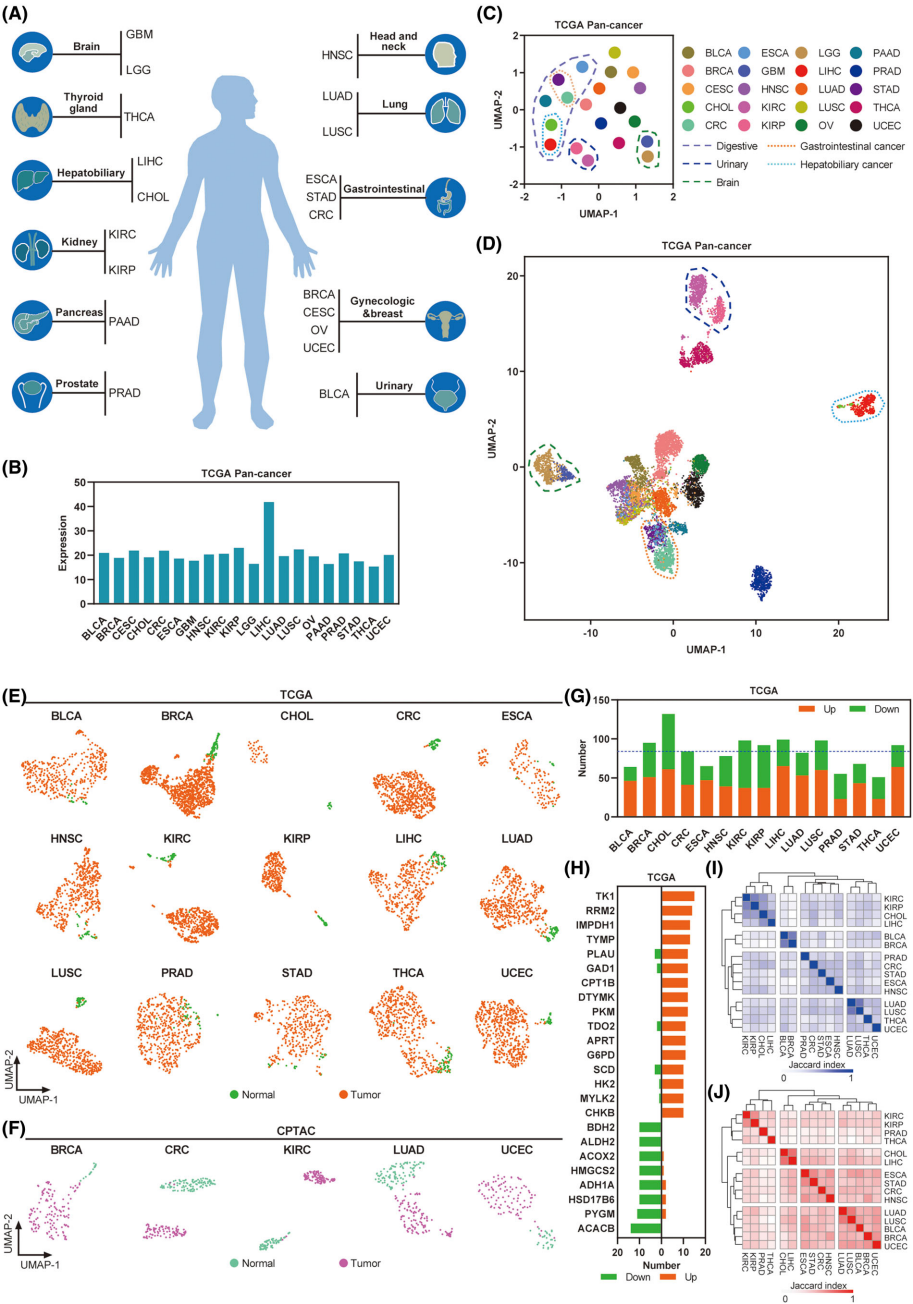
The global transcriptome patterns of metabolic rate‐limiting enzymes across human cancers: (A) Schematic drawing of the TCGA cancer types were used in this study; (B) expression of 168 metabolic rate‐limiting enzymes in TCGA human cancers; (C) Uniform Manifold Approximation and Projection (UMAP) plot showing the similarities in expression of the metabolic rate‐limiting enzymes among TCGA cancer types. See Table S1 for abbreviations of each TCGA cancer type; (D) UMAP plot showing the individual differences in the genome‐wide global expression profiles among the TCGA cancer types in individual cases; (E) UMAP plot showing the expression of metabolic rate‐limiting enzymes in TCGA cancer types with tumour and normal cases; (F) similar to (E), but in CPTAC protein level datasets; (G) the number of significantly dysregulated metabolic rate‐limiting enzymes for each cancer from the TCGA; (H) bar plot showing metabolic rate‐limiting enzymes that were upregulated/downregulated in more than 10 TCGA cancer types; (I and J) heat map visualizing the matrix of the Jaccard indices of the shared connections for the upregulated (I) and downregulated (J) metabolic rate‐limiting enzymes of each cancer from the TCGA

We focused our analysis on 20 cancer types with primary solid tumours in the TCGA database (Figure 1A and Table S1). The expression abundance of 168 metabolic rate‐limiting enzymes was nearly identical across the included cancers, except for LIHC, which showed the highest levels (Figure 1B and Table S2). Next, the connectivity among these cancer types was evaluated using UMAP for dimension reduction based on the expression patterns of these metabolic enzymes (Figure 1C). Interestingly, we found a relationship in expression patterns among cancer types that shared similar tissue types of origins (Figure 1C). For instance, digestive system cancer types, such as ESCA, STAD, CRC, LIHC, CHOL and PAAD, were closely clustered (Figure 1C). Urinary system cancers (KIRC and KIRP) and brain tumours (GBM and LGG) were similar to digestive system cancers (Figure 1C).

To determine the expression patterns of these enzymes in individual tumours, UMAP was used to visualize the global expression patterns for more than 8000 individual tumour samples of 20 TCGA cancer types. We found marked overlap in expression among individuals with different cancer types that contain a large fraction of squamous cells, such as HNSC, LUSC and CESC (Figure 1D). Some gastrointestinal (CRC and STAD), hepatobiliary (CHOL and LIHC), urinary system (KIRC and KIRP) and brain (GBM and LGG) cancer types showed relatively unique global expression patterns of these enzymes that had little overlap with other cancer types (Figure 1D). We also used UMAP in cancer cell lines from the DepMap Pan‐cancer dataset, which showed similar results for gastrointestinal (CRC and STAD) and brain (GBM and LGG) cancer types (Figure S1). These results indicate that metabolic rate‐limiting enzymes expression can vary considerably among individual tumours of different system types. However, global expression patterns are similar within cases in the same system cancer type.

### 3.2 Metabolic rate‐limiting enzymes are frequently dysregulated across human cancers

Next, we analysed 15 cancer types, each of which harboured at least 6 normal samples in the TCGA dataset. The connectivity between tumour and normal tissue samples was evaluated using UMAP based on the expression of these enzymes in each cancer type. We found that tumours were well separated from the normal samples in BRCA, CRC, CHOL, KIRP, KIRC, LIHC, LUSC, LUAD and UCEC (Figure 1E). We obtained similar results in an analysis of five cancer types from the CPTAC‐released protein expression dataset (Figure 1F and Table S3). Next, a series of metabolic rate‐limiting enzymes were significantly dysregulated (fold change >1.5/ < .66 and *p* < .05) in each TCGA cancer type, accounting for 27%–71% of all enzymes (Figure 1G). We found that 123 or 113 enzymes showed consistent upregulation or downregulation in at least 2 cancer types (Figure 1G), respectively. Interestingly, 16 or 8 metabolic rate‐limiting enzymes were upregulated or downregulated, respectively, in more than half of the cancer types (Figure 1H), suggesting that these metabolic rate‐limiting enzymes may be functionally conserved. To investigate the connectivity of dysregulated metabolic rate‐limiting enzymes among these cancer types, the Jaccard indices were computed to evaluate the overlap of their dysregulated enzymes. We observed that cancers with similar tissue types of origins were clustered together, such as gastrointestinal (CRC and STAD), hepatobiliary (CHOL and LIHC) and urinary system cancers (KIRC and KIRP) (Figure 1I,J), suggesting that they have common features, which is consistent with the abovementioned findings. Collectively, our analyses indicated that metabolic rate‐limiting enzymes are frequently dysregulated across human cancers.

### 3.3 Genomic alterations of metabolic rate‐limiting enzymes across human cancers

**FIGURE 2:**
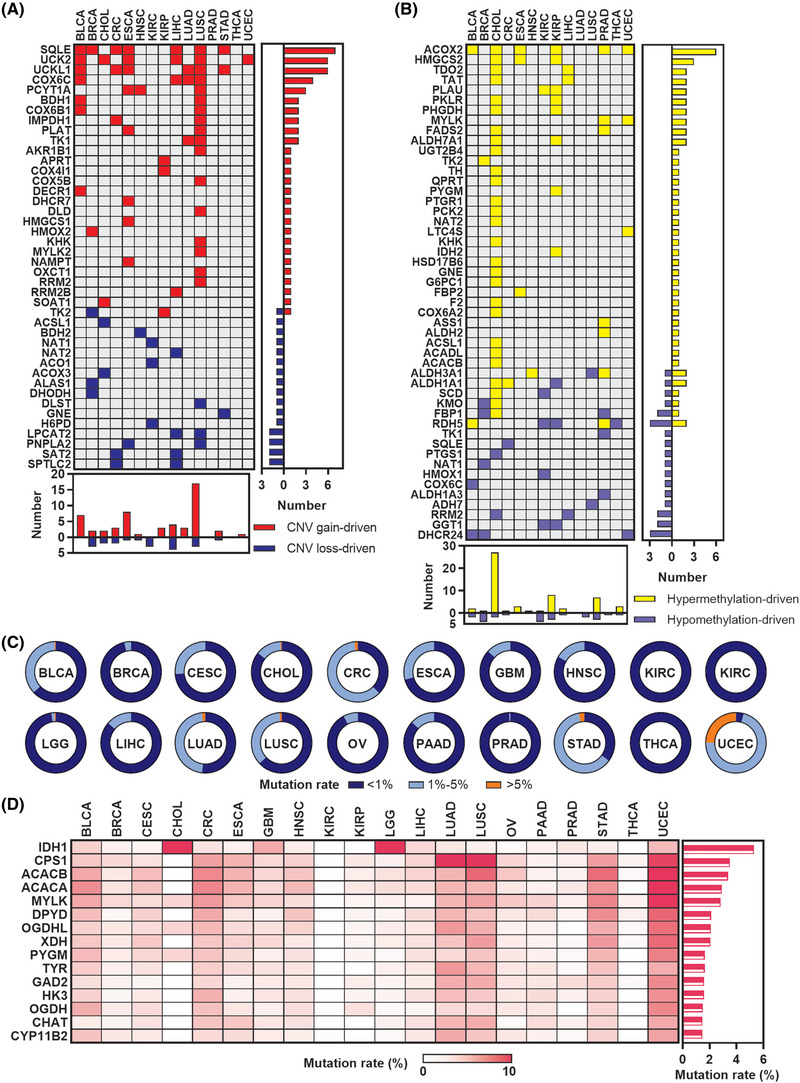
Genomic alterations of metabolic rate‐limiting enzymes in human cancers: (A) plot showing the 42 copy number variations (CNV)‐driven metabolic rate‐limiting enzymes that presented in at least one cancer type from TCGA; (B) plot showing the 49 hypermethylation‐/hypomethylation‐driven metabolic rate‐limiting enzymes that presented in at least 1 cancer type from TCGA; (C) pie charts showing the proportion of metabolic rate‐limiting enzymes mutation rates in each cancer from the TCGA; (D) heat map showing the top 15 mutant metabolic rate‐limiting enzymes in TCGA Pan‐cancer cases

To explore the genomic features of these enzymes in cancers, we first analysed the CNV and DNA methylation alterations that affect the expression of genes related to tumourigenesis and development. We found that 42 CNV‐driven metabolic rate‐limiting enzymes (upregulation of gene expression due to CNV gain in tumours; downregulation of gene expression due to CNV loss in tumours) were present in at least one cancer type, and LUSC had the most CNV‐driven genes (*N* = 20, Figure 2A). We also found 49 hypermethylation‐/hypomethylation‐driven metabolic rate‐limiting enzymes (downregulation of gene expression due to hypermethylation in tumours; upregulation of gene expression due to hypomethylation in tumours) that were present in at least one cancer type, and CHOL had the most hypermethylation‐/hypomethylation‐driven genes (*N* = 29, Figure 2B). These analyses suggest that some metabolic rate‐limiting enzymes may be affected by changes in CNV or DNA methylation levels in human cancers. Next, we constructed a mutational landscape of these enzymes in human cancers. Most cancer types showed lower mutation rates in metabolic rate‐limiting enzymes, except UCEC (Figure 2C). Although some metabolic rate‐limiting enzymes (i.e. IDH1 and CPS1) demonstrated higher mutation rates in parts of cancer types, most metabolic rate‐limiting enzymes had lower mutation rates in Pan‐cancer human cancers (Figure 2D). Our results revealed that part of the dysregulated metabolic rate‐limiting enzymes is caused by the genomic or epigenetic alteration in human cancers.

### 3.4 Metabolic rate‐limiting enzymes demonstrate extensive clinically relevant patterns

**FIGURE 3:**
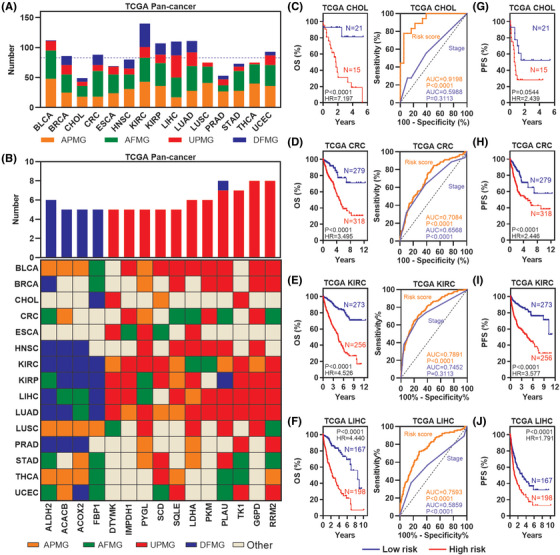
Identification of dysregulated metabolic rate‐limiting enzymes with clinical relevance: (A) bar plot showing the prognostic metabolic rate‐limiting enzymes across multiple cancers from the TCGA; (B) plot showing DFMGs/UPMGs that in at least five cancer types; (C–F) Kaplan–Meier plot (left) showing the Overall survival (OS) of the risk score; ROC plot (right) showing the AUC of the risk score and TNM stage in CHOL (C), colorectal cancer (CRC) (D), KIRC (E) and LIHC (F) in TCGA; (G–J) Kaplan–Meier plot showing the PFS of the risk score in CHOL (G), CRC (H), KIRC (I) and LIHC (J) in TCGA

To comprehensively identify clinically relevant metabolic rate‐limiting enzymes, a series of analyses were performed to identify potential prognostic metabolic rate‐limiting enzymes that are associated with patient survival time. Four types of prognostic enzymes were defined: enzymes associated with poor/favourable OS (APMG/AFMG), upregulated enzymes associated with poor OS (UPMG) and downregulated enzymes associated with poor OS (DFMG). Prognostic metabolic rate‐limiting enzymes comprised 29%–83% of all enzymes (Figure 3A). Moreover, 4 DFMGs (ALDH2, ACACB, ACOX2 and FBP1) and 11 UPMGs (DTYMK, IMPDH1, PYGL, SCD, SQLE, LDHA, PKM, PLAU, TK1, G6PD and RRM2) were present in at least 5 cancer types, and these 15 enzymes were also APMG or AFMG in at least 1 cancer type (Figure 3B), which suggests tumour specificity and functional conservation of these metabolic rate‐limiting enzymes.

To further explore the potential effect of prognostic metabolic rate‐limiting enzymes, COX regression models were used to generate a risk score based on the expression of UPMG/DFMG and OS in four cancer types. Our analyses showed that a high‐risk score (>0) was significantly associated with poor survival in CHOL, CRC, KIRC and LIHC (Figure 3C‐F, left). Moreover, the risk score based on the gene expression of UPMG/DFMG had a more significant prognostic effect than TNM stage in these four cancer types (AUC range .708‐.919, Figure 3C‐F, right). Additionally, a high‐risk score (>0) also predicted poor PFS in these cancer types, although the *p*‐value for CHOL was .054 (Figure 3G‐J). These results indicate that our models based on the expression of UPMG/DFMG are good indicators of survival and prognosis in CHOL, CRC, KIRC and LIHC.

### 3.5 Metabolic rate‐limiting enzymes are associated with Myc in human cancers

**FIGURE 4:**
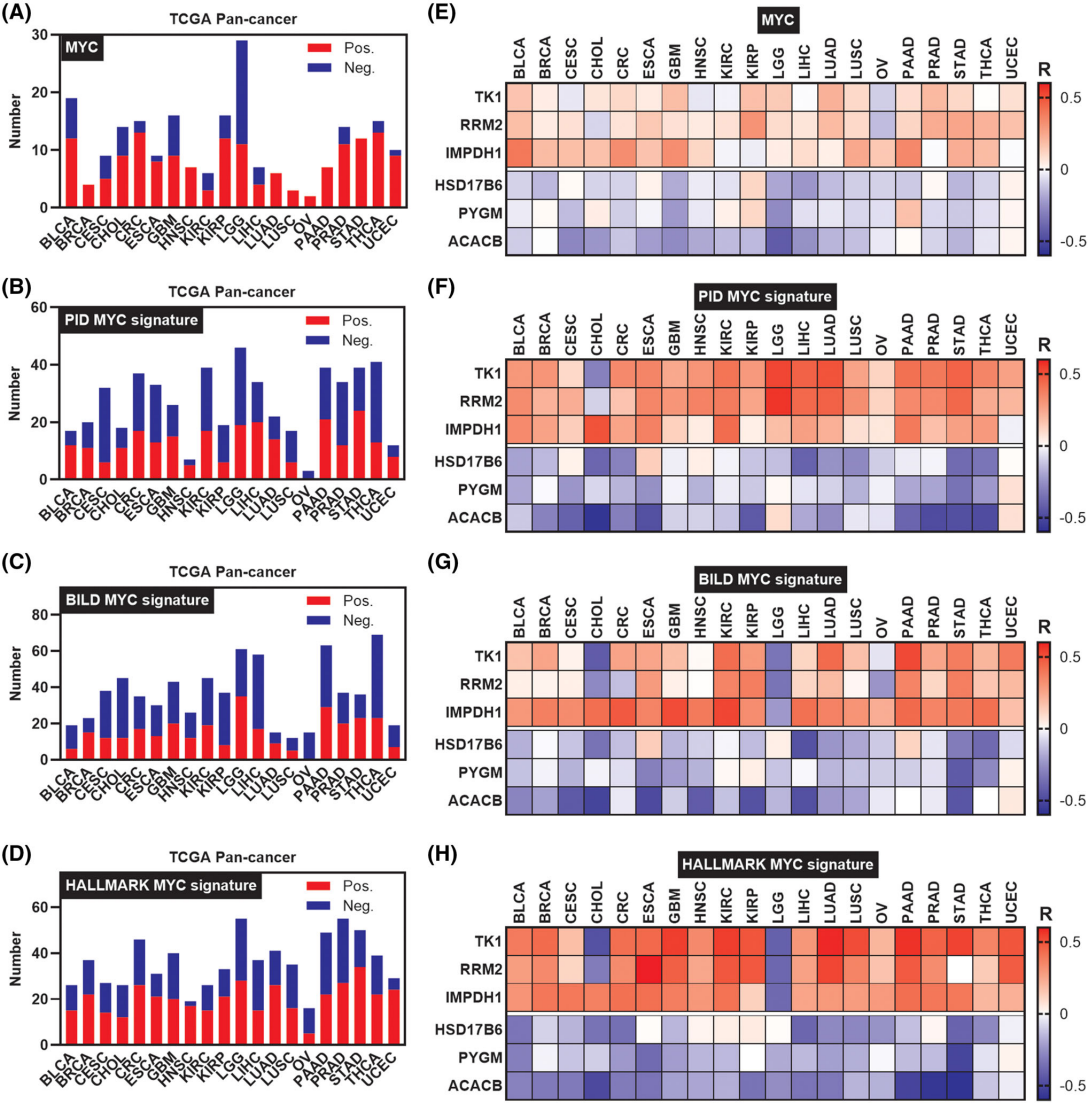
Correlations between Myc/Myc signatures and the transcriptome landscape of metabolic rate‐limiting enzymes in human cancers: (A) bar plot showing the number of Myc‐correlated metabolic rate‐limiting enzymes for each human cancer from the TCGA; (B–D) bar plots showing the number of PID (B), BILD (C) and HALLMARK (D) Myc signatures‐correlated metabolic rate‐limiting enzymes for each human cancer from the TCGA. Pos. represents *R* > .25 and *p* < .05; Neg. represents *R*< .25 and *p* < .05; (E) heat map showing the correlations between Myc expression and the top three of upregulated/downregulated rate‐limiting enzymes in TCGA cancers; (F–H) heat maps showing the correlations between PID (F), BILD (G) and HALLMARK (H) Myc signatures and the top three of upregulated/downregulated rate‐limiting enzymes in TCGA cancers

Myc is a major transcription factor that is frequently deregulated and highly expressed in at least 50% of cancers. Previous studies have shown that Myc reprograms cancer cell metabolism to promote cell growth.^28^, ^29^, ^30^, ^31^ We first analysed the correlation between Myc and these enzymes. In half of the cancer types, more than 10 enzymes were correlated with Myc at the transcriptional level based on TCGA dataset (Figure 4A). To further explore the relationship between Myc and these enzymes, we used three independent Myc signatures and found that over 75% of the cancer types had more than 20% enzymes that were significantly correlated with at least one Myc signature (|*R*| > .25 and *p* < .05) (Figure 4B–D). More than 25% of these enzymes were correlated with at least one Myc signature in four digestive system cancer types, including CHOL, CRC, LIHC and STAD (Figure 4B–D). We identified 16 upregulated metabolic rate‐limiting enzymes and 8 downregulated metabolic rate‐limiting enzymes in more than half of the cancer types in the abovementioned analysis (Figure 1H). Thus, we analysed the correlation of the top three upregulated enzymes (TK1, RRM2 and IMPDH1) and downregulated metabolic rate‐limiting enzymes (HSD17B6, PYGM and ACACB) with Myc/Myc signatures. Impressively, TK1, RRM2 and IMPDH1 were positively correlated with Myc/Myc signatures in multiple cancer types, whereas HSD17B6, PYGM and ACACB were negatively correlated with Myc/Myc signatures (Figure 4E–H). These findings revealed that a series of metabolic rate‐limiting enzymes are associated with Myc/Myc signatures, particularly in digestive system cancer types. The top three upregulated and downregulated enzymes are positively and negatively correlated with Myc/Myc signatures, respectively.

### 3.6 IMPDH1 is a prognostic metabolic rate‐limiting enzyme that is upregulated in CRC

**FIGURE 5:**
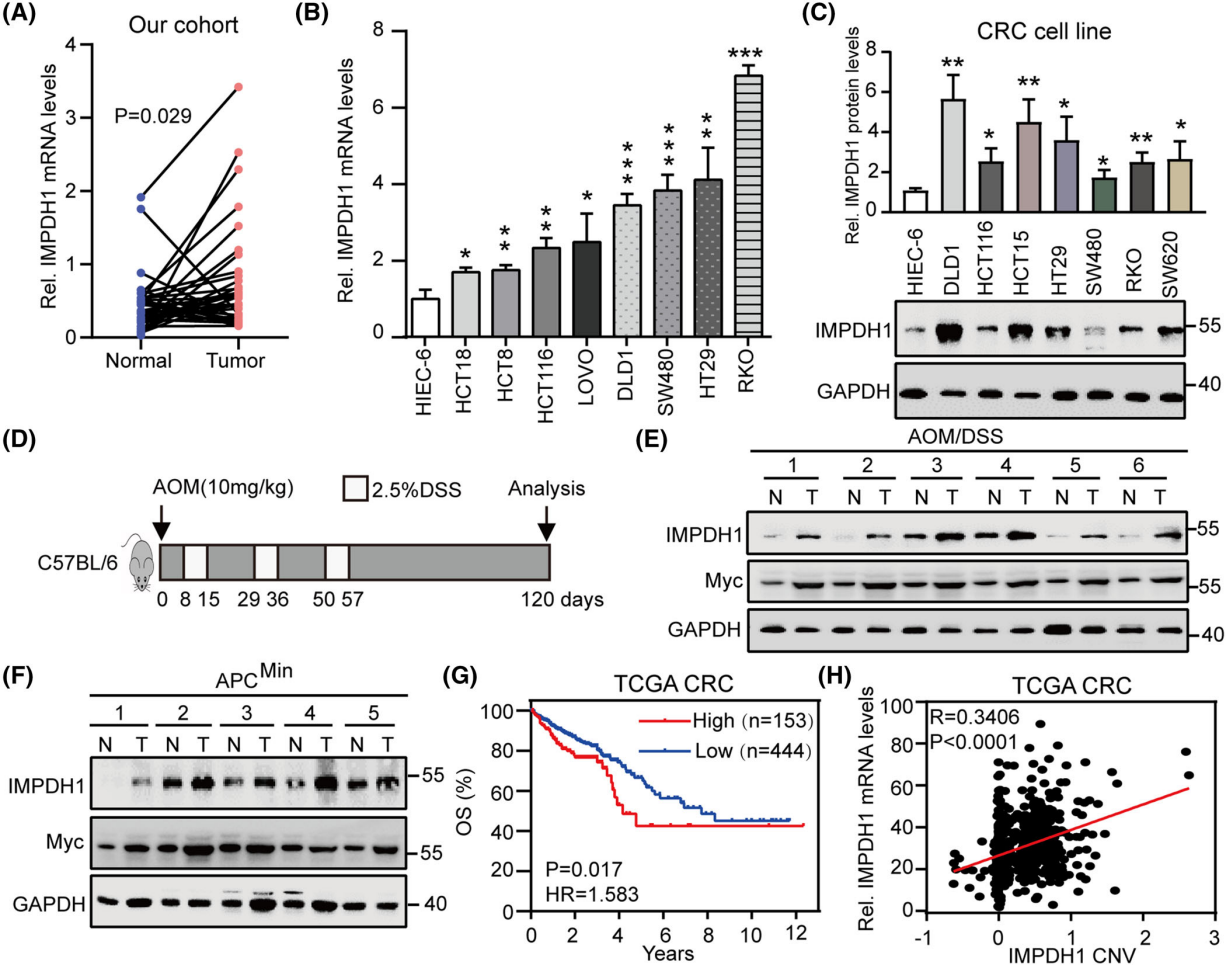
Identify inosine monophosphate dehydrogenase 1 (IMPDH1) as a prognostic metabolic rate‐limiting enzyme that is upregulated in colorectal cancer (CRC): (A) analyses of IMPDH1 mRNA expression in adjacent normal tissues versus primary tumour samples from The Sixth Affiliated Hospital of Sun Yat‐sen University; (B) analyses of IMPDH1 mRNA expression in eight CRC cell lines and normal intestinal epithelial cells HIEC6; (C) analyses of IMPDH1 protein expression in seven CRC cell lines and normal intestinal epithelial cells HIEC6; (D) schematic diagram showing the experimental design for azoxymethane/dextran sulphate sodium (AOM/DSS)‐induced mouse CRC model; (E) expression of IMPDH1 and Myc protein in normal colon tissues (N) and paired tumour samples (T) from AOM/DSS‐inducted CRC mice analysed by immunoblotting; (F) expression of IMPDH1 and Myc protein in normal small intestinal tissues (N) and intestinal adenoma samples (T) from APC^Min/+^ mouse (21 weeks of age) by immunoblotting; (G) Kaplan–Meier overall survival curves of human CRC patients with low versus high IMPDH1 mRNA in TCGA CRC dataset; (H) positive correlation between IMPDH1 expression and IMPDH1 copy number variations in TCGA CRC dataset. Data are presented as mean ± SD; *p < .05, **p < .01, ***p < .001

CRC showed a variety of specificities in the abovementioned results; we, therefore, selected six CRC‐dysregulated metabolic rate‐limiting enzymes (IMPDH1, MYLK, XDH, DPYD, UGDH and PTGS1) for validation in our clinical cohort by qRT‐PCR assays (Figures 5A, S2A and Table S4). Particularly, IMPDH1 is upregulated in more than half of cancer types and associated with poor survival, as well as positively correlated with Myc/Myc signatures in human cancers according to our abovementioned results. Moreover, the role of IMPDH1 has not yet been further reported in CRC. Hence, IMPDH1 was selected for subsequent analyses. We observed that IMPDH1, but not the related gene IMPDH2, was significantly overexpressed in all 15 cancers (Figure S2B), implying the key role of IMPDH1 in tumourigenesis. To further evaluate the expression of IMPDH1 in CRC, qRT‐PCR assays were performed in eight CRC cell lines and normal intestinal epithelial cells HIEC6. The results showed that IMPDH1 is significantly overexpressed in CRC cell lines (Figure 5B). Moreover, the analysis of the additional CRC cohorts indicated that IMPDH1, but not the related gene IMPDH2, is associated with CRC progression (Figure S2C).

In addition to the upregulation of IMPDH1 transcription, fuller analysis showed that IMPDH1 protein was also markedly upregulated in CRC (Figures 5C andS2D). Furthermore, C57BL/6 mice were treated with azoxymethane/dextran sulphate sodium to induce CRC (which occurred by 17 weeks), and western blotting showed that IMPDH1 protein was highly expressed in CRC (Figure 5D,E). Interestingly, high IMPDH1 protein expression was also observed in spontaneous intestinal tumours from APC^Min+^ mice (Figure 5F). Additionally, an analysis of OS suggested that poorer survival of CRC patients with high IMPDH1 levels (Figure 5G). Genetic analysis showed that the upregulation of IMPDH1 mRNA was markedly correlated with IMPDH1 CNV gain (Figure 5H). Collectively, these findings suggest that IMPDH1 is highly expressed at both transcriptional and protein levels and is associated with poor survival in CRC patients.

### 3.7 The Myc–IMPDH1/2 axis promotes CRC growth by increasing de novo GTP biosynthesis

**FIGURE 6:**
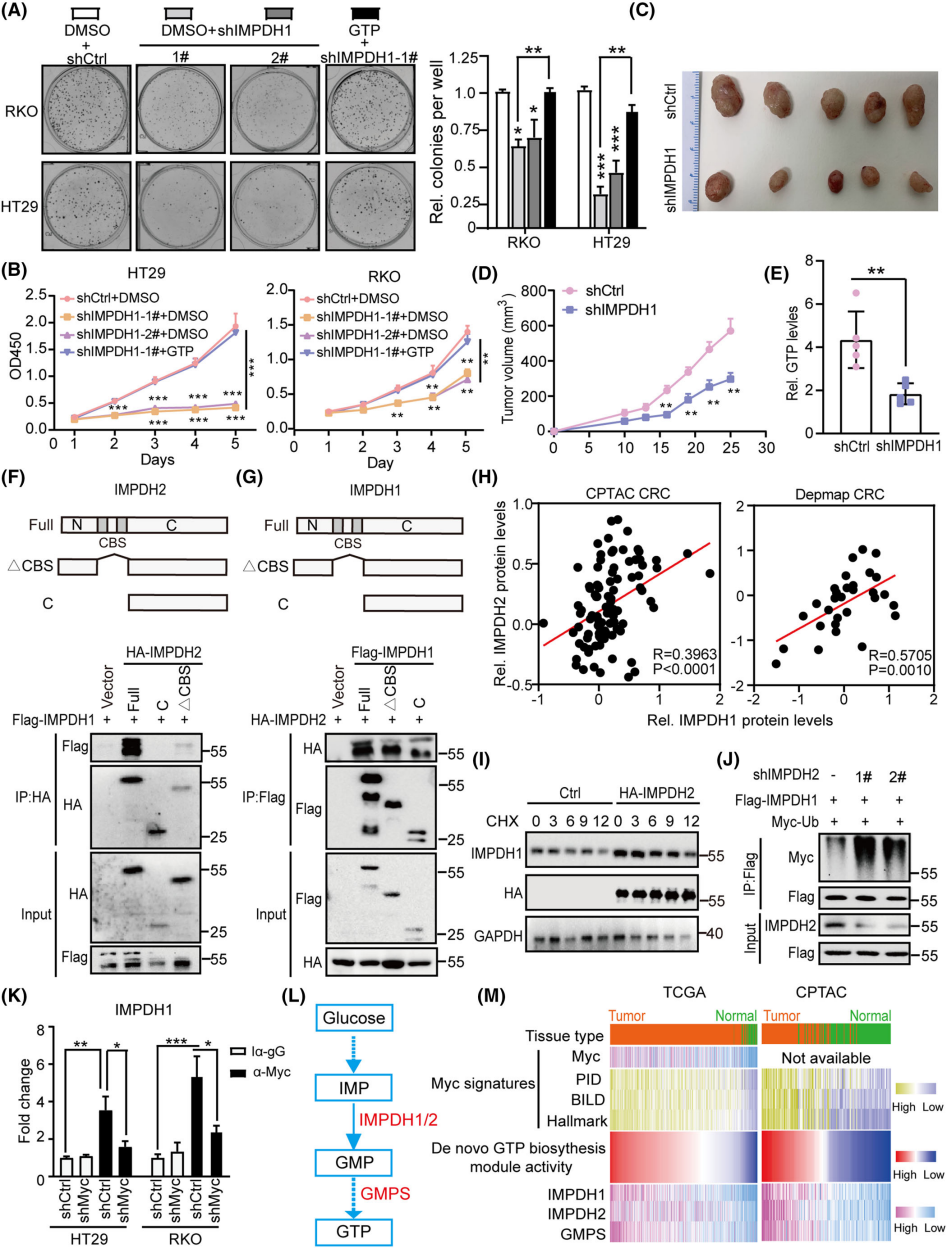
The Myc–IMPDH1/2 axis promotes colorectal cancer (CRC) growth by regulating GTP metabolic reprogramming: (A) colony formation of RKO and HT29 cells stably expressing the indicated vectors or treated with GTP (100 μM) (left), and bar graphs showing the colony numbers (right); (B) CCK‐8 assays were performed in RKO and HT29 cells stably expressing the indicated plasmids or treated with GTP (100 μM); (C) tumour photographs from subcutaneous xenograft; *n* = 5, per group; (D) subcutaneous xenograft experiments were performed in HT29 cells stably expressing the indicated plasmids; (E) analyses of GTP levels in tumour tissues from subcutaneous xenograft experiments; (F and G) inosine monophosphate dehydrogenase 1 (IMPDH1)–IMPDH2 interaction via N‐terminal and C‐terminal domains. Generation of IMPDH2‐mutant constructs (F) and IMPDH1‐mutant constructs (G). HEK293T cell lysates transfected with indicated plasmids analysed for co‐immunoprecipitation (Co‐IP); (H) correlation analysis between IMPDH1 and IMPDH2, based on CPTAC and DepMap CRC datasets; (I) time‐course analysis of IMPDH1 protein levels in IMPDH2‐overexpressed HEK293T cells; (J) IMPDH2 depletion increases the polyubiquitination levels of IMPDH1. HEK293T cells were transfected with the indicated plasmids, treated with MG132 for 6 h and then lysed in lysis buffer. Immunoprecipitation of ubiquitin‐conjugated IMPDH1 proteins with anti‐FLAG affinity agarose and subjected to immunoblot assay with Myc tag antibody; (K) Myc occupancy on the IMPDH1 promoters. Chromatin immunoprecipitation (ChIP) was performed with the endogenous Myc antibody in HT29 and RKO cells transfected with shCtrl or shMyc plasmid. qPCR analysis was performed on the endogenous promoters of IMPDH1 gene; (L) schematic diagram of the GTP biosynthesis pathway; (M) heat maps showing correlations between Myc expression level and Myc signatures and de novo GTP biosynthesis module activity in the TCGA and CPTAC CRC datasets. Data are presented as mean ± SD; *p < .05, **p < .01. ***p < .001

Although IMPDH2 promotes tumourigenesis by regulating de novo GTP synthesis,^15^, ^23^ the role of the related protein IMPDH1 in tumourigenesis is not fully understood. To explore the role of IMPDH1 in CRC tumourigenesis, we silenced IMPDH1 using specific short hairpin RNAs (shRNAs) in two CRC cell lines (RKO and HT29). Colony formation experiments and CCK‐8 assays suggested that IMPDH1 depletion inhibited CRC cell colony formation and proliferation, whereas the phenotype was rescued by GTP addition (Figure 6A,B). To confirm whether IMPDH1 promotes CRC growth in vivo, IMPDH1‐knock‐down HT29 cells were subcutaneously implanted into the flanks of nude mice. Mice injected with IMPDH1‐knock‐down HT29 cells showed lower GTP synthesis and tumour growth rates than those injected with control cells (Figures 6C–E andS3A,B). These results reveal that IMPDH1 promotes CRC growth by increasing GTP synthesis.

Unexpectedly, we identified IMPDH2 as a binding partner of IMPDH1 in HEK293T and HCT116 cells by analysing Huttlin's mass spectrometry data.^32^ Co‐IP assays confirmed the interaction between IMPDH1 and IMPDH2 (Figure 6F,G). Further analyses showed that the N‐terminal domain of IMPDH2 was responsible for its binding to IMPDH1, whereas the C‐terminal domain of IMPDH1 was required for its interaction with IMPDH2 (Figure 6F,G). Interestingly, correlation analysis showed a positive correlation between the IMPDH1 and IMPDH2 proteins (Figure 6H), implying a possible regulatory relationship between IMPDH1 and IMPDH2. Western blot assay showed that the depletion or overexpression of IMPDH1 did not alter the protein abundance of IMPDH2, whereas overexpression of IMPDH2 significantly increased the protein abundance of IMPDH1 (Figure S3C‐F). The result of protein half‐life assay showed that overexpression of IMPDH2 significantly extended the half‐life of endogenous IMPDH1 (Figure 6I). In addition, in vivo ubiquitination experiments showed that the depletion of IMPDH2 increased the polyubiquitination of IMPDH1, whereas IMPDH2 overexpression has the opposite effects (Figures 6J and S3G). These results suggested that IMPDH2 promotes IMPDH1 protein stability by decreasing the polyubiquitination levels of IMPDH1, which synergistically increases the de novo GTP biosynthesis to promote tumourigenesis.

Given the correlation between Myc and metabolic rate‐limiting enzymes (Figure 4) and the relationship between Myc and IMPDH1 or IMPDH2 in CRC cell lines (Figure S3H), we hypothesized that Myc promotes de novo GTP biosynthesis by regulating the expression of IMPDH1 and IMPDH2 in CRC. As expected, overexpression of Myc increased the mRNA and protein levels of IMPDH1 and IMPDH2, whereas Myc knock‐down has the opposite effects, suggesting that Myc regulates the expression of IMPDH1 and IMPDH2 (Figure S3I–K). Chromatin immunoprecipitation experiments showed Myc binding at the IMPDH1 and IMPDH2 promoter regions (Figures 6K and S3L,M). As expected, Myc depletion inhibited CRC cell colony formation and proliferation, whereas the phenotype was rescued by GTP addition (Figure S3N–P). Furthermore, a significant positive correlation among Myc expression, Myc signatures and de novo GTP biosynthesis module activity was also found in TCGA and CPTAC CRC cohorts (Figure 6L,M). These findings suggest that the Myc–IMPDH1/2 axis promotes CRC growth by increasing de novo GTP biosynthesis.

### 3.8 The Myc–IMPDH1/2 axis is dysregulated across human cancers

**FIGURE 7:**
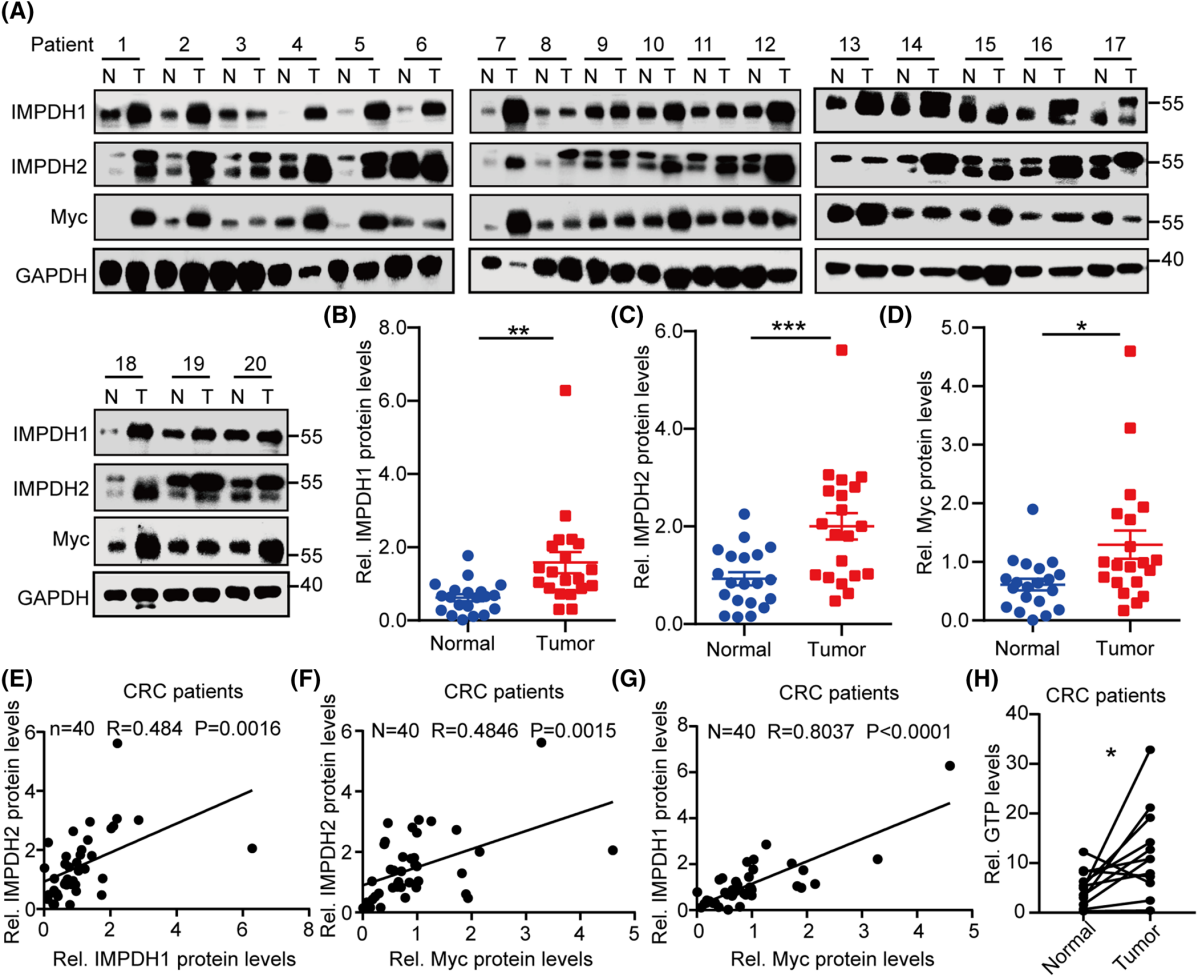
The Myc–IMPDH1/2 axis is dysregulated in human cancers: (A) immunoblot analyses of the indicated proteins in human colorectal cancers (CRCs) from The Sixth Affiliated Hospital of Sun Yat‐sen University; (B–D) relative protein levels of inosine monophosphate dehydrogenase 1 (IMPDH1) (B), IMPDH2 (C) and Myc (D). The proteins were quantified by densitometry from (A), with GAPDH as a normalizer; (E) correlation analysis of IMPDH1 and IMPDH2 proteins from (A); (F) correlation analysis of Myc and IMPDH1 proteins from (A); (G) correlation analysis of Myc and IMPDH2 proteins from (A); (H) analyses of GTP levels in adjacent normal tissues versus matched CRC tumour tissues. Data are presented as mean ± SD; *p < .05, **p < .01

To confirm whether the Myc–IMPDH1/2 axis is dysregulated in cancers, we performed a single‐sample gene set enrichment analysis in human cancers. We observed the dysregulation of the Myc–IMPDH1/2 axis in eight cancers, including CRC (Figure S4). To better evaluate the dysregulation of Myc–IMPDH1/2 axis in CRC, 20 pairs of CRC tumour (T) and adjacent normal colon tissue (N) samples were analysed by immunoblotting (Figure 7A). We observed that Myc, IMPDH1 and IMPDH2 protein levels were markedly increased in CRC tissues compared to those in normal colon samples (Figure 7B–D). Moreover, the positive correlations between Myc and IMPDH1, Myc and IMPDH2 and IMPDH1 and IMPDH2 were confirmed in our CRC samples (Figure 7E–G). More importantly, GTP levels were higher in CRC samples than in the paired normal samples (Figure 7H). Taken together, these data support the idea that the Myc–IMPDH1/2 axis plays a crucial role in tumourigenesis and suggests IMPDH1/2 as viable targets for cancer treatment.

## 4 DISCUSSION

**FIGURE 8:**
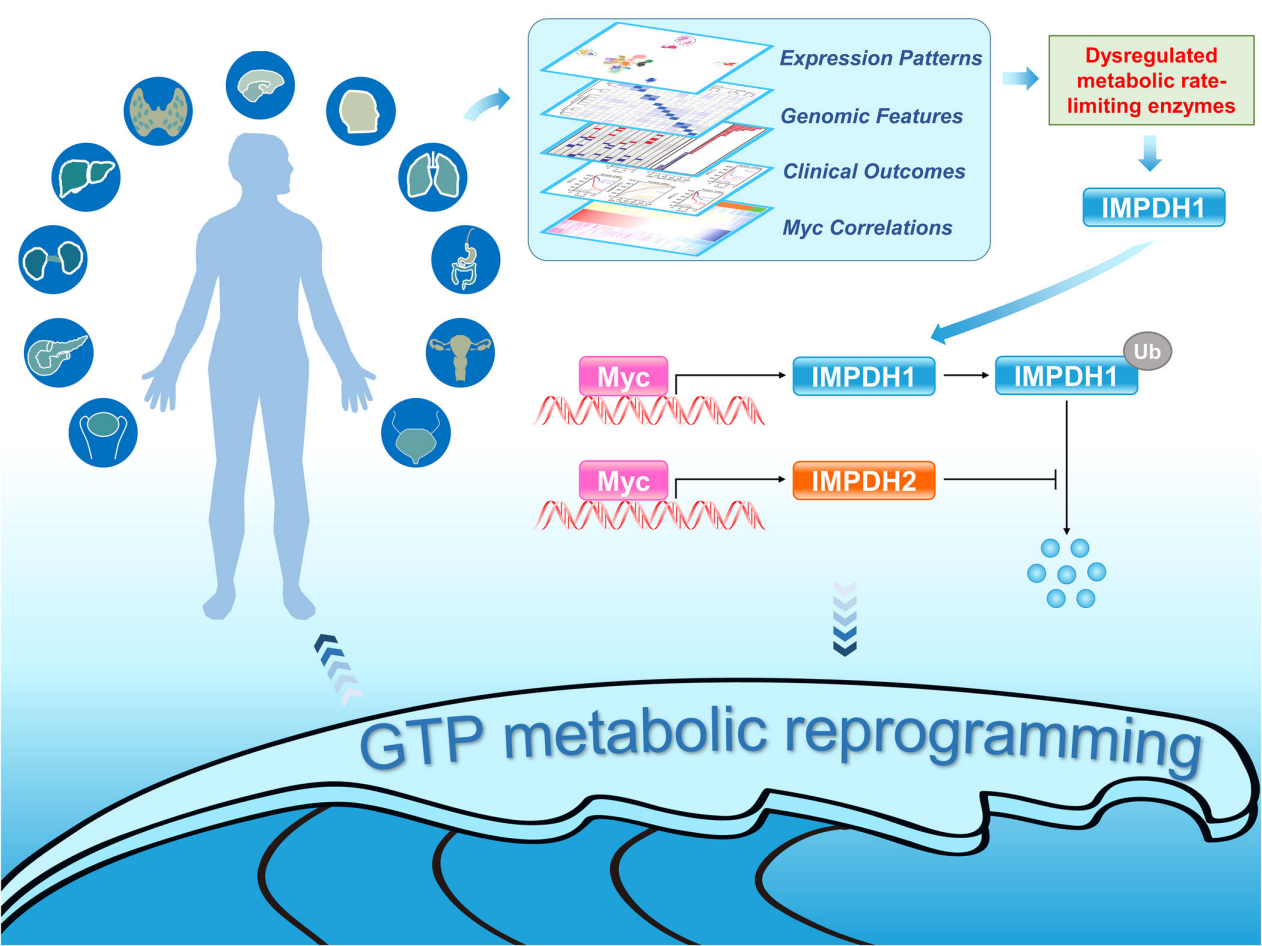
Schematic summary shows that Myc–IMPDH1/2 axis promotes tumourigenesis by regulating GTP metabolic reprogramming

In this study, we found similar global expression patterns of metabolic rate‐limiting enzymes for cancers of similar tissue origin, despite interindividual variation within each cancer type, which reveals that targeting metabolic rate‐limiting enzymes may be general treatment for cancers of similar tissue origin. Our comprehensive Pan‐cancer study revealed that these enzymes are dysregulated in various cancers and are associated with Myc/Myc signatures, particularly IMPDH1. We further experimentally demonstrated that IMPDH2 promotes IMPDH1 protein stability by decreasing the polyubiquitination levels of IMPDH1, and the Myc–IMPDH1/2 axis promotes tumourigenesis by regulating GTP metabolic reprogramming (Figure 8). These findings suggest that the inhibition of the Myc–IMPDH1/2 axis or IMPDH1 may be a viable option for cancer treatment, especially CRC.

Metabolic reprogramming caused by the dysregulation of metabolic enzymes plays an important role in tumourigenesis.^33^, ^34^, ^35^, ^36^, ^37^ However, metabolic rate‐limiting enzymes, the most important enzymes involved in cell metabolism, have not been systematically analysed in human cancers. In this study, we performed a comprehensive and systematic analysis and provided landscapes of these enzymes in diverse cancer types. A previous Pan‐cancer study demonstrated that various protein‐coding genes are differentially expressed in cancer, and individual liver cancer tumours showed relatively unique global expression patterns and little overlap with other cancer types.^38^ Interestingly, the expression patterns of metabolic rate‐limiting enzymes are not only unique to hepatobiliary cancer types (LIHC and CHOL) but also show relatively unique expression patterns in gastrointestinal cancer types (CRC and STAD), brain tumours (GBM and LGG) and kidney cancers (KIRP and KIRC). Our findings extend this previous Pan‐cancer research. Our data suggest that future study should be further classified based on biomedical functions of human protein‐coding genes to fully understand the implication of its expression patterns. We also identified a series of metabolic rate‐limiting enzymes related to genomic alteration and prognosis, such as SQLE and FBP1, which were reported as CNV‐expression correlated genes and played important roles in human cancers.^39^, ^40^, ^41^ Nonetheless, these genomic alterations and prognosis‐related enzymes need to be further validated in future study to explore their roles in tumourigenesis.

Oncogenic Myc drives metabolic reprogramming through the transcriptional regulation of metabolic enzymes.^8^, ^9^, ^10^, ^11^, ^12^, ^13^, ^14^ However, researchers have not yet established a connection between metabolic rate‐limiting enzymes and Myc. In this study, we established a link between the 168 metabolic rate‐limiting enzymes and Myc and identified the dysregulation of the Myc–IMPDH1/2 axis in human cancers. These results show the crosstalk between Myc and the metabolic rate‐limiting enzymes, complementing our knowledge of Myc‐related metabolic reprogramming.

IMPDH2 is thought to be more abundant than IMPDH1 in most tissues,^15^, ^23^ which led to the role of IMPDH1 in tumourigenesis being largely ignored. Here, we found that IMPDH1, but not the related gene IMPDH2, was significantly overexpressed in 15 cancer types (Figure S2B). In particular, we observed that the fold change in IMPDH1 expression in tumours compared to normal tissues was significantly higher than the fold change in IMPDH2 expression in CRC (Table S4), implying a pivotal role for IMPDH1 in tumourigenesis. Furthermore, we found that IMPDH2 interacts with IMPDH1 and promotes the stability of IMPDH1 protein, which deepens our understanding of IMPDH‐associated de novo GTP biosynthesis. Although we found IMPDH1 stabilizes IMPDH2 by reducing the polyubiquitination levels of IMPDH1, the molecular mechanism has not been identified. We will conduct follow‐up research in the future.

In conclusion, our study illustrates the importance of metabolic rate‐limiting enzymes, and the crosstalk between Myc and these enzymes in human cancers. In particular, IMPDH2 stabilizes IMPDH1 by reducing the polyubiquitination levels of IMPDH1, Myc transcriptionally activates IMPDH1/2 by binding to the IMPDH1/2 promoter region and the Myc–IMPDH1/2 axis promotes tumourigenesis by regulating GTP metabolic reprogramming. Our findings proposed that the inhibition of the Myc–IMPDH1/2 axis or IMPDH1 may be a viable option for cancer treatment, especially CRC.

## CONFLICTS OF INTEREST

The authors declare that they have no conflicts of interest.

## Supporting information

Supporting Information

Supporting Information
