# Supplementary material for: c‐Myc–IMPDH1/2 axis promotes tumourigenesis by regulating GTP metabolic reprogramming
Source: Clin Transl Med. 2023 Jan 11;13(1):e1164. doi: 10.1002/ctm2.1164 (PMC9832425; doi:10.1002/ctm2.1164)
Supplement: Supplementary file 1 — Supporting Information [file CTM2-13-e1164-s001.docx]

**c-Myc-IMPDH1/2 axis promotes tumorigenesis by regulating GTP metabolic reprogramming**

Qiang Zhang, Kaisa Cui, Xiaoya Yang, Qilang He, Jing Yu, Li Yang, Gang Yao, Weiwei Guo, Zhanhao Luo, Yugeng Liu, Yuan Chen, Zhen He, Ping Lan

**Supplementary Materials and Methods**

- 1. **Differential expression analysis**

Uniform Manifold Approximation and Projection (UMAP) analyses were performed using R software 4.1.0 with the umap package. For the Figure1c analysis, we used UMAP to cluster TCGA Pan-cancer samples based on mean expression value of each metabolic rate-limiting enzyme in each cancer type. Dysregulated metabolic rate-limiting enzymes were identified as previously described ^1^. 15 cancer types compassed tumor and normal samples that over five cases. Dysregulated rate-limiting enzymes were identified as previously described: fold change >1.5 and *P* < 0.05 were considered as upregulated genes, while change <0.66 and *P* < 0.05 were considered as downregulated gene^1^. The Jaccard index was used to evaluate the ratio of the dysregulated metabolic rate-limiting enzymes that are common to two cancers. Graphpad prism 9 and the Pheatmap package of R software 4.1.0 were used to analyze and visualize hierarchical cluster analyses.

- 1. **CNV, DNA Methylation and Mutation Analyses**

Three criteria were made to identify CNV gain/loss-driven metabolic rate-limiting enzymes for each cancer type: (1) upregulated/downregulated genes with more than 40% tumor samples with CNV > 0.1/< -0.1; (2) the mean values of CNV in tumor samples > |0.1| (3) Pearson correlation between expression and CNV > 0.3, false discovery rate (FDR) < 0.05 was considered significant. Besides, we made two criteria to discover DNA hypermethylation/hypomethylation-driven dysregulation of metabolic rate-limiting enzymes: (1) difference methylation levels (β-value) of the genes between tumor and normal samples >|0.05| for each cancer type. FDR<0.05 were retained; (2) Pearson correlations between gene expression levels and methylation levels <−0.3 and FDR<0.05. STAD was the absence of normal samples data with DNA methylation level. The R software 4.1.0 was used to analyze the Pearson correlations between gene expression and CNV/methylation levels. The Maftools package from R software 4.1.0 was used to analyze mutation across human cancers from the TCGA^2^. Nonsynonymous mutations were included in this study, and silent variants were excluded.

- 1. **Survival analysis**

Overall survival (OS) analyses were performed as previously described**:** cases were divided into high and low expression groups for each metabolic rate-limiting enzyme gene, differences in *P* value were examined in the OS of the groups according to a Kaplan–Meier survival analysis, the value yielding the lowest log-rank p-values from the 10th to 90th percentiles of the samples was selected^1, 3^. Hazard ratio (HR) > 1 and P<0.05 in the five-year were considered significantly associated with poor survival, while 0 < HR < 1 and P < 0.05 were regarded to be associated with favorable survival. Cox regression model analyses were performed using Graphpad prism 9. The risk score was generated by Z-score transformed linear predictor value from COX regression model analysis based on the expression of clinically relevant enzyme genes in related cancer types.

- 1. **Enrichment analysis**

Three Myc signatures were obtained from the Molecular Signatures Database (http://www.gsea-msigdb.org/gsea/msigdb/genesets.jsp). Myc signature levels, de novo GTP biosynthesis levels and Myc-IMPDH1/2 axis levels were calculated based on ssGSEA package in R software 4.1.0.

- 1. **AOM/DSS-induced mouse CRC model**

Azoxymethane/dextran sulfate sodium (AOM/DSS)-induced mouse CRC model was performed as described previously^4^. Briefly, eight-week-old C57BL/6 mice were intraperitoneally injected with 10 mg/kg AOM (Sigma-Aldrich), and given drinking water containing 2.5% DSS (MP Biomedicals, Santa Ana, CA, USA) for one week, followed by regular drinking water for two weeks. The mice were fed with 2.5% DSS water for two rounds for one week and sacrificed on the 120th day. Tumor and normal tissues from mouse colon were harvested for immunoblotting.

**References**

1. Cui K, Liu C, Li X, Zhang Q, Li Y. Comprehensive characterization of the rRNA metabolism-related genes in human cancer. *Oncogene*. 2020;39:786-800.

2. Mayakonda A, Lin DC, Assenov Y, Plass C, Koeffler HP. Maftools: efficient and comprehensive analysis of somatic variants in cancer. *Genome Res*. 2018;28:1747-1756.

3. Cui K, Yao S, Zhang H, et al. Identification of an immune overdrive high-risk subpopulation with aberrant expression of FOXP3 and CTLA4 in colorectal cancer. *Oncogene*. 2021;40:2130-2145.

4. Parang B, Barrett CW, Williams CS. AOM/DSS Model of Colitis-Associated Cancer. *Methods Mol Biol*. 2016;1422:297-307.

**Supplementary figures**

**
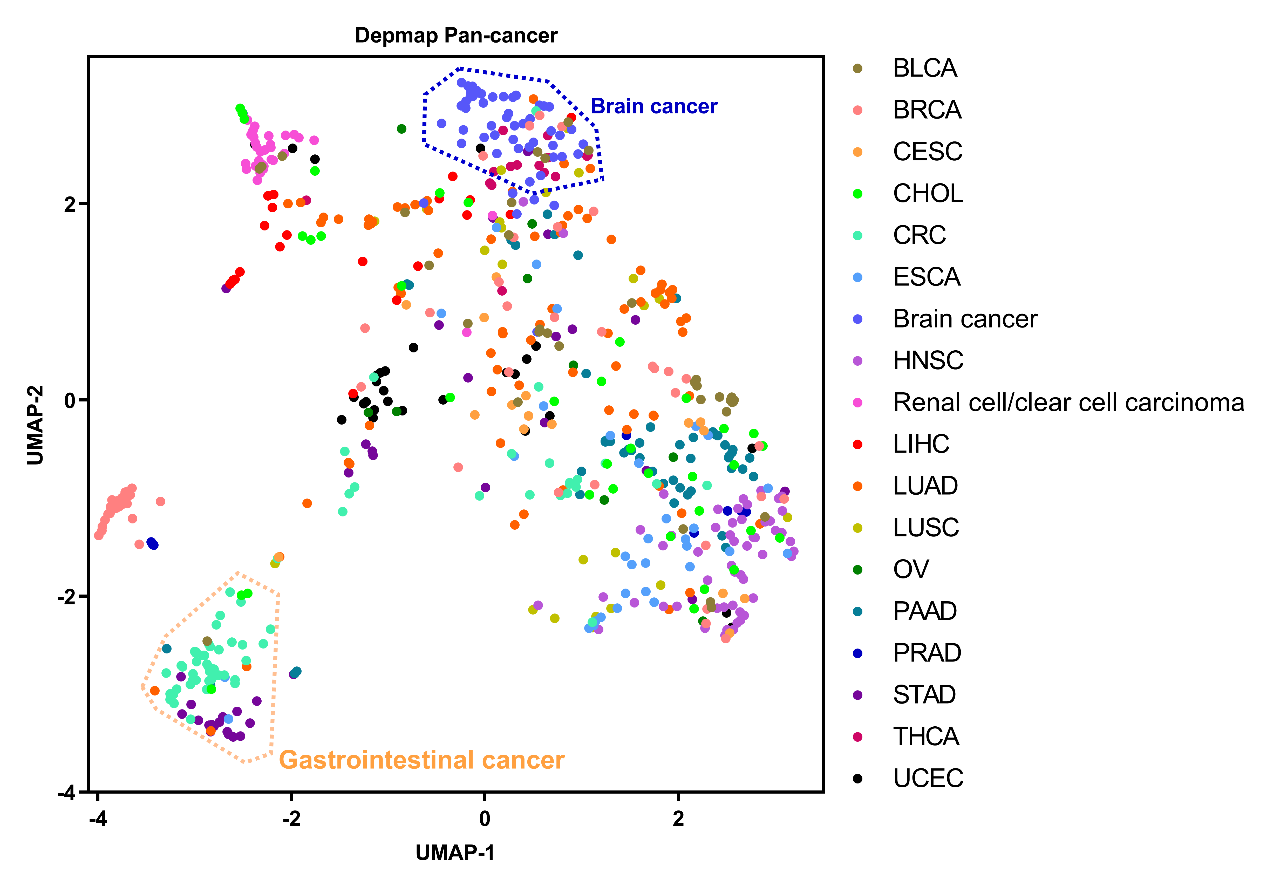
**

**Figure S1** UMAP plot showing the individual differences in the genome-wide global expression profiles among the Depmap Pan-cancer data in individual cancer cell lines.


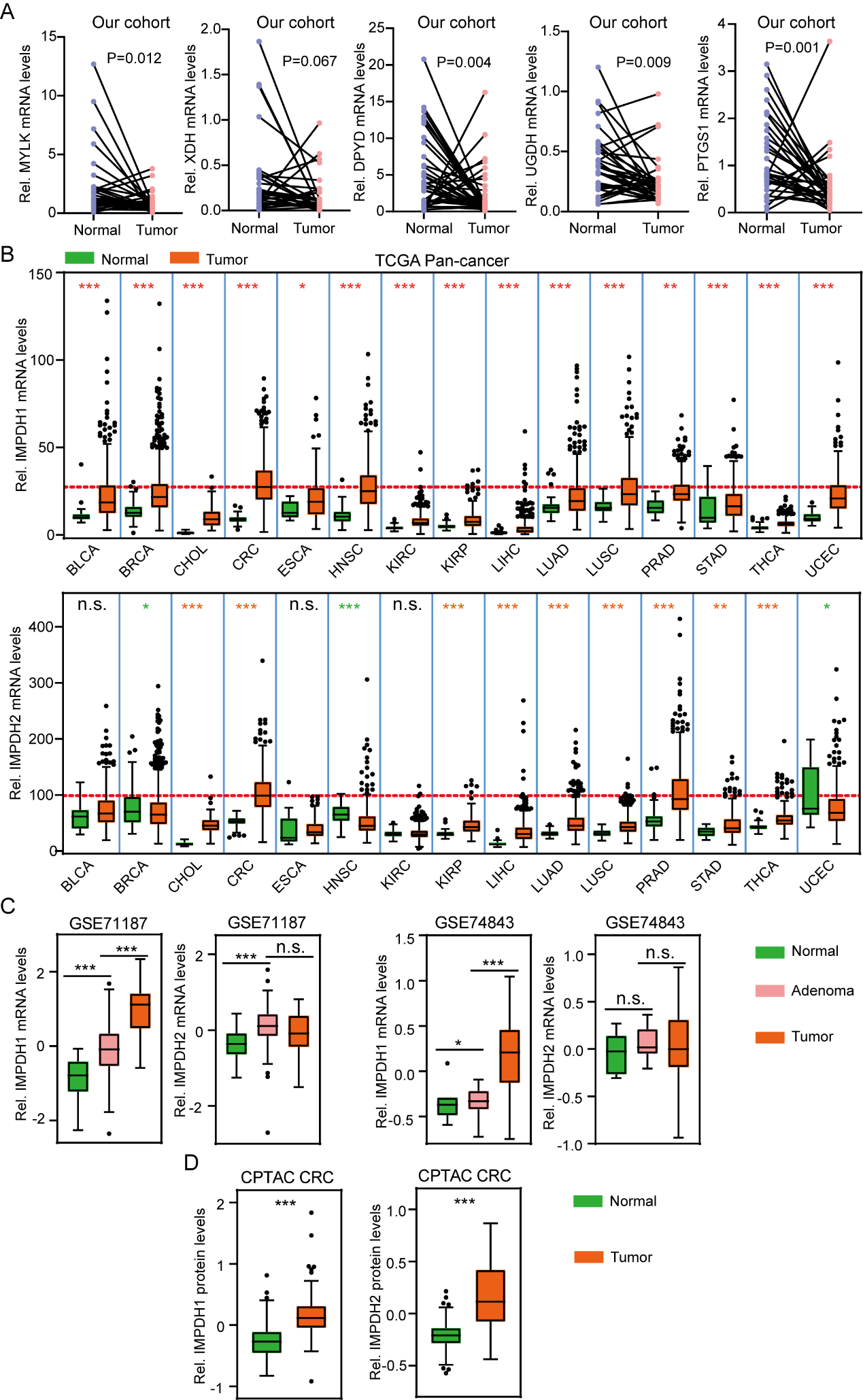


**Figure S2** **IMPDH1 is upregulated in tumor.**

**A** The analysis of MYLK, XDH, DPYD, UGDH, and PTGS1 mRNA expression in adjacent normal tissues versus primary tumor samples from The Sixth Affiliated Hospital of Sun Yat-sen University. **B** IMPDH1 (not related gene IMPDH2) is significantly overexpressed in 15 cancers. **C** IMPDH1 (not related gene IMPDH2) is associated with CRC progression. **D** Analysis of IMPDH1 and IMPDH2 protein expression in CPTAC dataset.

Data are presented as mean ± SD; * *P* < 0.05, ** *P* < 0.01, *** *P* < 0.001.


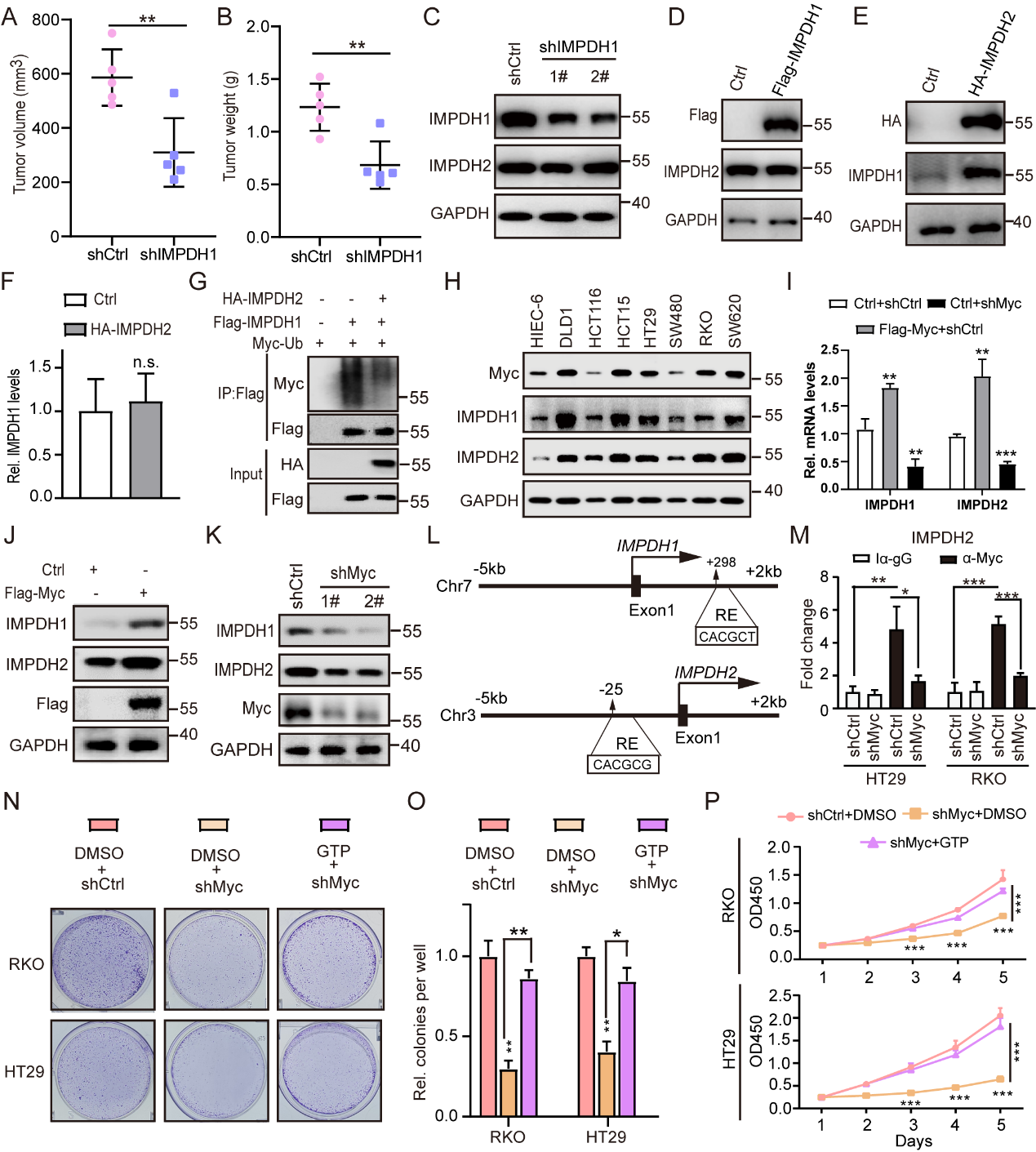


**Figure S3** **The Myc-IMPDHs axis promotes CRC growth.**

**A-B** Decreased tumor volume (**A**) and tumor weight (**B**) by IMPDH1 knockdown in subcutaneous xenograft. **C-****D** IMPDH1 knockdown or overexpression does not affect IMPDH2 protein abundance. HEK293T cells were transfected with IMPDH1 shRNAs (**C**) or Flag-IMPDH1 (**D**) plasmids and cell lysates were analyzed for immunobloting. **E** IMPDH2 overexpression increased IMPDH1 protein abundance. HEK293T cells were transfected with HA-IMPDH2 plasmids and cell lysates were analyzed for immunobloting. **F** IMPDH2 did not affect IMPDH1 transcription. HEK293T cells transfected with vetor or HA-IMPDH2 plasmids were analyzed for RT-qPCR. **G** IMPDH2 overexpression decreases the polyubiquitination levels of IMPDH1. HEK293T cells were transfected with the indicated plasmids, treated with MG132 for 6 h and then lysed in Lysis buffer. Immuno-precipitation of ubiquitin-conjugated IMPDH1 proteins with anti-FLAG affinity agarose and subjected to immuno-blot assay with Myc tag antibody. **H** Expression of Myc, IMPDH1, and IMPDH2 in HIEC6 and CRC cell lines. HIEC6 and CRC cell lines were lysed for immunobloting. **I** Myc promotes IMPDH1/2 expression. HEK293T cells transfected with indicated plasmids were analyzed for RT-qPCR. **J** Myc overexpression increases IMPDH1/2 protein abundance. HEK293T cells were transfected with Flag-Myc plasmids and cell lysates were analyzed for immunobloting. **K** Myc depletion decreases IMPDH1/2 protein abundance. HEK293T cells were transfected with indicated plasmids and cell lysates were analyzed for immunobloting. **L** Schematic presentation of Myc binding element on the IMPDH1/2 locus. RE, responsive element. **M** Myc occupancy on the IMPDH2 promoters. ChIP was performed with the endogenous Myc antibody in HT29 and RKO cells transfected with shCtrl or shMyc plasmid. qPCR analysis was performed on the endogenous promoters of IMPDH2 gene. **N-O** Colony formation of RKO and HT29 cells stably expressing the indicated vectors or treated with GTP (100 μM) (**N**), and bar graphs showing the colony numbers (**O**). **P** CCK-8 assays were performed in RKO and HT29 cells stably expressing the indicated plasmids or treated with GTP (100 μM).

Data are presented as mean ± SD; * *P* < 0.05, ** *P* < 0.01, *** *P* < 0.001.


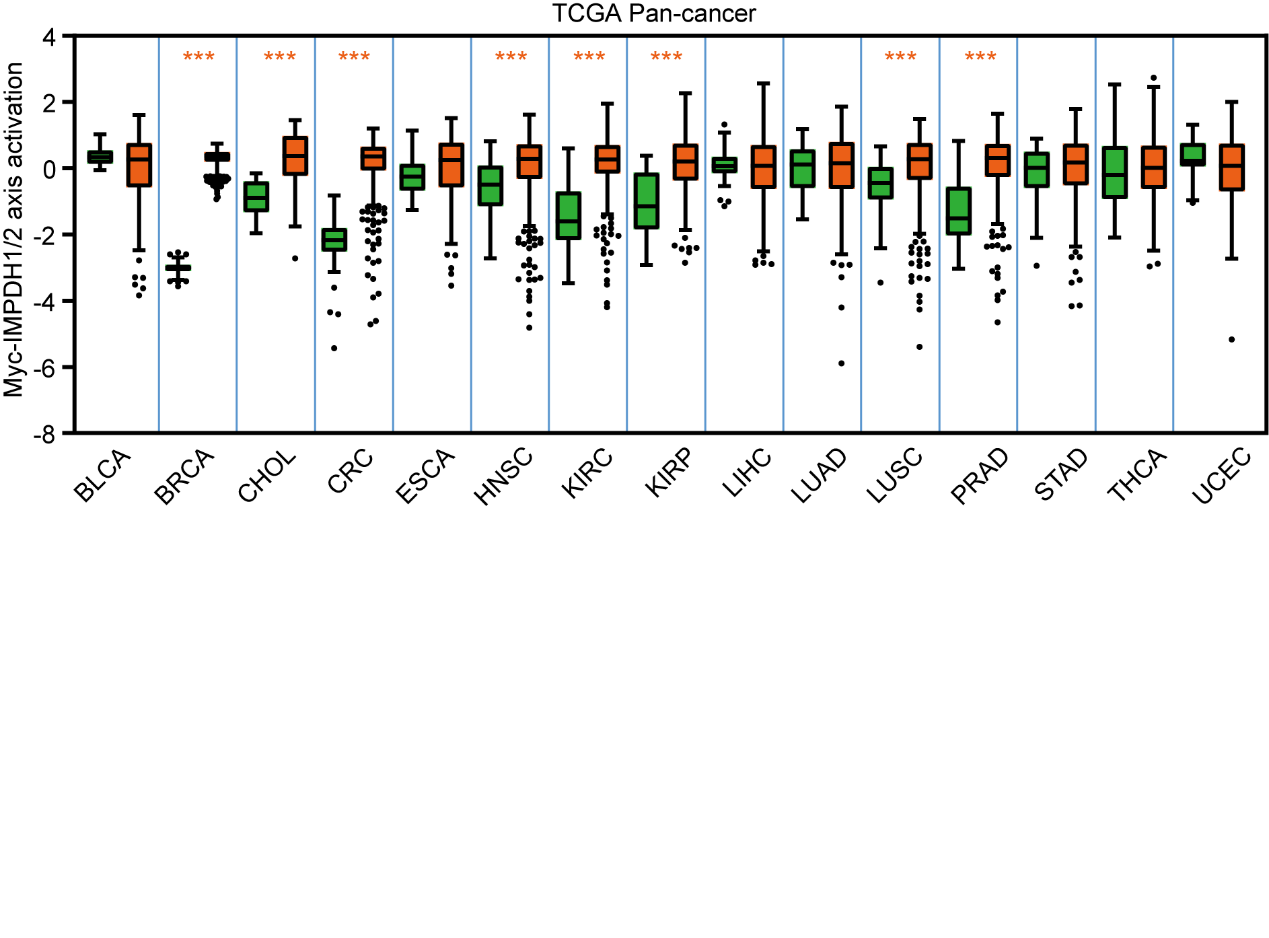


**Figure S4** The Myc-IMPDHs axis is overexpressed in human cancers.
