# Supplementary material for: c‐Myc–IMPDH1/2 axis promotes tumourigenesis by regulating GTP metabolic reprogramming
Source: Clin Transl Med. 2023 Jan 11;13(1):e1164. doi: 10.1002/ctm2.1164 (PMC9832425; doi:10.1002/ctm2.1164)
Supplement: Supplementary file 2 — Supporting Information [file CTM2-13-e1164-s002.docx]

**Table.S1 List of TCGA cancer types.**

| **Project (Abbreviations)** | **Cancer Type** | **Primary Site** | **Tumor** | **Normal** |
| --- | --- | --- | --- | --- |
|  |  |  |  |  |
|  |  |  |  |  |
| **BLCA** | Bladder Urothelial Carcinoma | Bladder | 408 | 19 |
| **BRCA** | Breast Invasive Carcinoma | Breast | 1091 | 113 |
| **CESC** | Cervical Squamous Cell Carcinoma and Endocervical Adenocarcinoma | Cervix | 304 | / |
| **CHOL** | Cholangiocarcinoma | Bile Duct | 36 | 9 |
| **CRC** | Colorectal Cancer | Colorectal | 625 | 51 |
| **ESCA** | Esophageal Carcinoma | Esophagus | 161 | 11 |
| **GBM** | Glioblastoma Multiforme | Brain | 154 | / |
| **HNSC** | Head and Neck Squamous Cell Carcinoma | Head and Neck | 502 | 44 |
| **KIRC** | Kidney Renal Clear Cell Carcinoma | Kidney | 531 | 72 |
| **KIRP** | Kidney Renal Papillary Cell Carcinoma | Kidney | 288 | 32 |
| **LGG** | Brain Lower Grade Glioma | Brain | 511 | / |
| **LIHC** | Hepatocellular Carcinoma | Liver | 373 | 50 |
| **LUAD** | Lung Adenocarcinoma | Lung | 513 | 59 |
| **LUSC** | Lung Squamous Cell Carcinoma | Lung | 501 | 49 |
| **OV** | Ovarian Serous Cystadenocarcinoma | Ovary | 374 | / |
| **PAAD** | Pancreatic Adenocarcinoma | Pancreas | 177 | / |
| **PRAD** | Prostate Adenocarcinoma | Prostate | 495 | 52 |
| **STAD** | Stomach Adenocarcinoma | Stomach | 375 | 32 |
| **THCA** | Thyroid Carcinoma | Thyroid | 502 | 58 |
| **UCEC** | Uterine Corpus Endometrial Carcinoma | Uterus | 543 | 35 |

**Table.S2 List of 168 metabolic rate-limiting enzymes.**

| **SYMBOL** | **GENENAME** | **ENTREZID** | **ENSEMBLID** | **Reference**  **(**PMID) |
| --- | --- | --- | --- | --- |
| ACACA | acetyl-CoA carboxylase alpha | 31 | ENSG00000278540 | 25965523 |
| ACACB | acetyl-CoA carboxylase beta | 32 | ENSG00000076555 | 25965523 |
| ACADL | acyl-CoA dehydrogenase long chain | 33 | ENSG00000115361 | 19468287 |
| ACADS | acyl-CoA dehydrogenase short chain | 35 | ENSG00000122971 | 32540485 |
| ACO1 | aconitase 1 | 48 | ENSG00000122729 | 19468287 |
| ACO2 | aconitase 2 | 50 | ENSG00000100412 | 19468287 |
| ACOX1 | acyl-CoA oxidase 1 | 51 | ENSG00000161533 | 29491006 |
| ACOX2 | acyl-CoA oxidase 2 | 8309 | ENSG00000168306 | 29535221 |
| ACOX3 | acyl-CoA oxidase 3, pristanoyl | 8310 | ENSG00000087008 | 29535221 |
| ACSL1 | acyl-CoA synthetase long chain family member 1 | 2180 | ENSG00000151726 | 19468287 |
| ADH1A | alcohol dehydrogenase 1A (class I), alpha polypeptide | 124 | ENSG00000187758 | 19468287 |
| ADH7 | alcohol dehydrogenase 7 (class IV), mu or sigma polypeptide | 131 | ENSG00000196344 | 19468287 |
| ADK | adenosine kinase | 132 | ENSG00000156110 | 19468287 |
| AKR1A1 | aldo-keto reductase family 1 member A1 | 10327 | ENSG00000117448 | 32566101 |
| AKR1B1 | aldo-keto reductase family 1 member B | 231 | ENSG00000085662 | 32566101 |
| ALAD | aminolevulinate dehydratase | 210 | ENSG00000148218 | 19468287 |
| ALAS1 | 5'-aminolevulinate synthase 1 | 211 | ENSG00000023330 | 19468287 |
| ALAS2 | 5'-aminolevulinate synthase 2 | 212 | ENSG00000158578 | 19468287 |
| ALDH1A1 | aldehyde dehydrogenase 1 family member A1 | 216 | ENSG00000165092 | 19468287 |
| ALDH1A2 | aldehyde dehydrogenase 1 family member A2 | 8854 | ENSG00000128918 | 19468287 |
| ALDH1A3 | aldehyde dehydrogenase 1 family member A3 | 220 | ENSG00000184254 | 19468287 |
| ALDH1B1 | aldehyde dehydrogenase 1 family member B1 | 219 | ENSG00000137124 | 19468287 |
| ALDH2 | aldehyde dehydrogenase 2 family member | 217 | ENSG00000111275 | 19468287 |
| ALDH3A1 | aldehyde dehydrogenase 3 family member A1 | 218 | ENSG00000108602 | 19468287 |
| ALDH3A2 | aldehyde dehydrogenase 3 family member A2 | 224 | ENSG00000072210 | 19468287 |
| ALDH7A1 | aldehyde dehydrogenase 7 family member A1 | 501 | ENSG00000164904 | 19468287 |
| ALDH9A1 | aldehyde dehydrogenase 9 family member A1 | 223 | ENSG00000143149 | 19468287 |
| ALOX5 | arachidonate 5-lipoxygenase | 240 | ENSG00000012779 | 19468287 |
| AMD1 | adenosylmethionine decarboxylase 1 | 262 | ENSG00000123505 | 33203990 |
| APRT | adenine phosphoribosyltransferase | 353 | ENSG00000198931 | 19468287 |
| ASS1 | argininosuccinate synthase 1 | 445 | ENSG00000130707 | 19468287 |
| BACE1 | beta-secretase 1 | 23621 | ENSG00000186318 | 19468287 |
| BDH1 | 3-hydroxybutyrate dehydrogenase 1 | 622 | ENSG00000161267 | 23082722 |
| BDH2 | 3-hydroxybutyrate dehydrogenase 2 | 56898 | ENSG00000164039 | 31819181 |
| CHAT | choline O-acetyltransferase | 1103 | ENSG00000070748 | 19468287 |
| CHKB | choline kinase beta | 1120 | ENSG00000100288 | 33058877 |
| COX4I1 | cytochrome c oxidase subunit 4I1 | 1327 | ENSG00000131143 | 19468287 |
| COX5B | cytochrome c oxidase subunit 5B | 1329 | ENSG00000135940 | 19468287 |
| COX6A1 | cytochrome c oxidase subunit 6A1 | 1337 | ENSG00000111775 | 19468287 |
| COX6A2 | cytochrome c oxidase subunit 6A2 | 1339 | ENSG00000156885 | 19468287 |
| COX6B1 | cytochrome c oxidase subunit 6B1 | 1340 | ENSG00000126267 | 19468287 |
| COX6C | cytochrome c oxidase subunit 6C | 1345 | ENSG00000164919 | 19468287 |
| CPS1 | carbamoyl-phosphate synthase 1 | 1373 | ENSG00000021826 | 33317798 |
| CPT1A | carnitine palmitoyltransferase 1A | 1374 | ENSG00000110090 | 30043753 |
| CPT1B | carnitine palmitoyltransferase 1B | 1375 | ENSG00000205560 | 31904482 |
| CPT2 | carnitine palmitoyltransferase 2 | 1376 | ENSG00000157184 | 33486313 |
| CS | citrate synthase | 1431 | ENSG00000062485 | 32284438 |
| CTPS1 | CTP synthase 1 | 1503 | ENSG00000171793 | 34129847 |
| CYP11B2 | cytochrome P450 family 11 subfamily B member 2 | 1585 | ENSG00000179142 | 32540969 |
| CYP7A1 | cytochrome P450 family 7 subfamily A member 1 | 1581 | ENSG00000167910 | 30122083 |
| DCK | deoxycytidine kinase | 1633 | ENSG00000156136 | 19468287 |
| DDC | dopa decarboxylase | 1644 | ENSG00000132437 | 19468287 |
| DECR1 | 2,4-dienoyl-CoA reductase 1 | 1666 | ENSG00000104325 | 32686647 |
| DGAT2 | diacylglycerol O-acyltransferase 2 | 84649 | ENSG00000062282 | 32829064 |
| DHCR24 | 24-dehydrocholesterol reductase | 1718 | ENSG00000116133 | 33422461 |
| DHCR7 | 7-dehydrocholesterol reductase | 1717 | ENSG00000172893 | 33422461 |
| DHODH | dihydroorotate dehydrogenase (quinone) | 1723 | ENSG00000102967 | 33249060 |
| DHPS | deoxyhypusine synthase | 1725 | ENSG00000095059 | 32235505 |
| DLD | dihydrolipoamide dehydrogenase | 1738 | ENSG00000091140 | 19468287 |
| DLST | dihydrolipoamide S-succinyltransferase | 1743 | ENSG00000119689 | 19468287 |
| DPYD | dihydropyrimidine dehydrogenase | 1806 | ENSG00000188641 | 19468287 |
| DTYMK | deoxythymidylate kinase | 1841 | ENSG00000168393 | 19468287 |
| F2 | coagulation factor II, thrombin | 2147 | ENSG00000180210 | 19468287 |
| FADS1 | fatty acid desaturase 1 | 3992 | ENSG00000149485 | 34438249 |
| FADS2 | fatty acid desaturase 2 | 9415 | ENSG00000134824 | 30923750 |
| FBP1 | fructose-bisphosphatase 1 | 2203 | ENSG00000165140 | 19468287 |
| FBP2 | fructose-bisphosphatase 2 | 8789 | ENSG00000130957 | 19468287 |
| G6PC1 | glucose-6-phosphatase catalytic subunit 1 | 2538 | ENSG00000131482 | 19468287 |
| G6PC2 | glucose-6-phosphatase catalytic subunit 2 | 57818 | ENSG00000152254 | 19468287 |
| G6PD | glucose-6-phosphate dehydrogenase | 2539 | ENSG00000160211 | 19468287 |
| GAD1 | glutamate decarboxylase 1 | 2571 | ENSG00000128683 | 19468287 |
| GAD2 | glutamate decarboxylase 2 | 2572 | ENSG00000136750 | 19468287 |
| GCH1 | GTP cyclohydrolase 1 | 2643 | ENSG00000131979 | 32980388 |
| GFPT1 | glutamine--fructose-6-phosphate transaminase 1 | 2673 | ENSG00000198380 | 34291417 |
| GFPT2 | glutamine-fructose-6-phosphate transaminase 2 | 9945 | ENSG00000131459 | 33846782 |
| GGT1 | gamma-glutamyltransferase 1 | 2678 | ENSG00000100031 | 19468287 |
| GLO1 | glyoxalase I | 2739 | ENSG00000124767 | 32858501 |
| GNE | glucosamine (UDP-N-acetyl)-2-epimerase/N-acetylmannosamine kinase | 10020 | ENSG00000159921 | 19468287 |
| GPAM | glycerol-3-phosphate acyltransferase, mitochondrial | 57678 | ENSG00000119927 | 19468287 |
| GPD2 | glycerol-3-phosphate dehydrogenase 2 | 2820 | ENSG00000115159 | 19468287 |
| H6PD | hexose-6-phosphate dehydrogenase/glucose 1-dehydrogenase | 9563 | ENSG00000049239 | 23167192 |
| HDC | histidine decarboxylase | 3067 | ENSG00000140287 | 19468287 |
| HK1 | hexokinase 1 | 3098 | ENSG00000156515 | 19468287 |
| HK2 | hexokinase 2 | 3099 | ENSG00000159399 | 19468287 |
| HK3 | hexokinase 3 | 3101 | ENSG00000160883 | 19468287 |
| HMGCR | 3-hydroxy-3-methylglutaryl-CoA reductase | 3156 | ENSG00000113161 | 19468287 |
| HMGCS1 | 3-hydroxy-3-methylglutaryl-CoA synthase 1 | 3157 | ENSG00000112972 | 19468287 |
| HMGCS2 | 3-hydroxy-3-methylglutaryl-CoA synthase 2 | 3158 | ENSG00000134240 | 19468287 |
| HMOX1 | heme oxygenase 1 | 3162 | ENSG00000100292 | 33923744 |
| HMOX2 | heme oxygenase 2 | 3163 | ENSG00000103415 | 33923744 |
| HSD17B6 | hydroxysteroid 17-beta dehydrogenase 6 | 8630 | ENSG00000025423 | 19468287 |
| HSD3B1 | hydroxy-delta-5-steroid dehydrogenase, 3 beta- and steroid delta-isomerase 1 | 3283 | ENSG00000203857 | 19468287 |
| HSD3B2 | hydroxy-delta-5-steroid dehydrogenase, 3 beta- and steroid delta-isomerase 2 | 3284 | ENSG00000203859 | 19468287 |
| IDH1 | isocitrate dehydrogenase (NADP(+)) 1 | 3417 | ENSG00000138413 | 33735687 |
| IDH2 | isocitrate dehydrogenase (NADP(+)) 2 | 3418 | ENSG00000182054 | 33735687 |
| IDO1 | indoleamine 2,3-dioxygenase 1 | 3620 | ENSG00000131203 | 30760888 |
| IDO2 | indoleamine 2,3-dioxygenase 2 | 169355 | ENSG00000188676 | 30760888 |
| IMPDH1 | inosine monophosphate dehydrogenase 1 | 3614 | ENSG00000106348 | 19468287 |
| IMPDH2 | inosine monophosphate dehydrogenase 2 | 3615 | ENSG00000178035 | 19468287 |
| KHK | ketohexokinase | 3795 | ENSG00000138030 | 32733884 |
| KMO | kynurenine 3-monooxygenase | 8564 | ENSG00000117009 | 30760888 |
| LDHA | lactate dehydrogenase A | 3939 | ENSG00000134333 | 34110068 |
| LIPE | lipase E, hormone sensitive type | 3991 | ENSG00000079435 | 19468287 |
| LPCAT2 | lysophosphatidylcholine acyltransferase 2 | 54947 | ENSG00000087253 | 19468287 |
| LTA4H | leukotriene A4 hydrolase | 4048 | ENSG00000111144 | 19468287 |
| LTC4S | leukotriene C4 synthase | 4056 | ENSG00000213316 | 19468287 |
| MYLK | myosin light chain kinase | 4638 | ENSG00000065534 | 19468287 |
| MYLK2 | myosin light chain kinase 2 | 85366 | ENSG00000101306 | 19468287 |
| MYLK3 | myosin light chain kinase 3 | 91807 | ENSG00000140795 | 19468287 |
| NAMPT | nicotinamide phosphoribosyltransferase | 10135 | ENSG00000105835 | 32111066 |
| NAT1 | N-acetyltransferase 1 | 9 | ENSG00000171428 | 19468287 |
| NAT2 | N-acetyltransferase 2 | 10 | ENSG00000156006 | 19468287 |
| OGDH | oxoglutarate dehydrogenase | 4967 | ENSG00000105953 | 19468287 |
| OGDHL | oxoglutarate dehydrogenase L | 55753 | ENSG00000197444 | 19468287 |
| OXCT1 | 3-oxoacid CoA-transferase 1 | 5019 | ENSG00000083720 | 32493060 |
| PAH | phenylalanine hydroxylase | 5053 | ENSG00000171759 | 19468287 |
| PCK1 | phosphoenolpyruvate carboxykinase 1 | 5105 | ENSG00000124253 | 19468287 |
| PCK2 | phosphoenolpyruvate carboxykinase 2, mitochondrial | 5106 | ENSG00000100889 | 19468287 |
| PCYT1A | phosphate cytidylyltransferase 1A, choline | 5130 | ENSG00000161217 | 20647050 |
| PCYT1B | phosphate cytidylyltransferase 1B, choline | 9468 | ENSG00000102230 | 20647050 |
| PEPD | peptidase D | 5184 | ENSG00000124299 | 34532344 |
| PFKM | phosphofructokinase, muscle | 5213 | ENSG00000152556 | 28176759 |
| PFKP | phosphofructokinase, platelet | 5214 | ENSG00000067057 | 33236133 |
| PHGDH | phosphoglycerate dehydrogenase | 26227 | ENSG00000092621 | 33284994 |
| PIK3C3 | phosphatidylinositol 3-kinase catalytic subunit type 3 | 5289 | ENSG00000078142 | 19468287 |
| PKLR | pyruvate kinase L/R | 5313 | ENSG00000143627 | 19468287 |
| PKM | pyruvate kinase M1/2 | 5315 | ENSG00000067225 | 19468287 |
| PLAT | plasminogen activator, tissue type | 5327 | ENSG00000104368 | 19468287 |
| PLAU | plasminogen activator, urokinase | 5328 | ENSG00000122861 | 19468287 |
| PNPLA2 | patatin like phospholipase domain containing 2 | 57104 | ENSG00000177666 | 33185110 |
| PTGR1 | prostaglandin reductase 1 | 22949 | ENSG00000106853 | 34421612 |
| PTGS1 | prostaglandin-endoperoxide synthase 1 | 5742 | ENSG00000095303 | 19468287 |
| PTGS2 | prostaglandin-endoperoxide synthase 2 | 5743 | ENSG00000073756 | 19468287 |
| PYGB | glycogen phosphorylase B | 5834 | ENSG00000100994 | 19468287 |
| PYGL | glycogen phosphorylase L | 5836 | ENSG00000100504 | 19468287 |
| PYGM | glycogen phosphorylase, muscle associated | 5837 | ENSG00000068976 | 19468287 |
| QPRT | quinolinate phosphoribosyltransferase | 23475 | ENSG00000103485 | 32390008 |
| RDH5 | retinol dehydrogenase 5 | 5959 | ENSG00000135437 | 19468287 |
| REN | renin | 5972 | ENSG00000143839 | 19468287 |
| RRM1 | ribonucleotide reductase catalytic subunit M1 | 6240 | ENSG00000167325 | 19468287 |
| RRM2 | ribonucleotide reductase regulatory subunit M2 | 6241 | ENSG00000171848 | 19468287 |
| RRM2B | ribonucleotide reductase regulatory TP53 inducible subunit M2B | 50484 | ENSG00000048392 | 19468287 |
| SAT1 | spermidine/spermine N1-acetyltransferase 1 | 6303 | ENSG00000130066 | 19468287 |
| SAT2 | spermidine/spermine N1-acetyltransferase family member 2 | 112483 | ENSG00000141504 | 19468287 |
| SCD | stearoyl-CoA desaturase | 6319 | ENSG00000099194 | 19468287 |
| SCD5 | stearoyl-CoA desaturase 5 | 79966 | ENSG00000145284 | 19468287 |
| SLC6A4 | solute carrier family 6 member 4 | 6532 | ENSG00000108576 | 33407815 |
| SOAT1 | sterol O-acyltransferase 1 | 6646 | ENSG00000057252 | 19468287 |
| SOAT2 | sterol O-acyltransferase 2 | 8435 | ENSG00000167780 | 19468287 |
| SPTLC1 | serine palmitoyltransferase long chain base subunit 1 | 10558 | ENSG00000090054 | 19468287 |
| SPTLC2 | serine palmitoyltransferase long chain base subunit 2 | 9517 | ENSG00000100596 | 19468287 |
| SQLE | squalene epoxidase | 6713 | ENSG00000104549 | 19468287 |
| STS | steroid sulfatase | 412 | ENSG00000101846 | 19468287 |
| TAT | tyrosine aminotransferase | 6898 | ENSG00000198650 | 19468287 |
| TDO2 | tryptophan 2,3-dioxygenase | 6999 | ENSG00000151790 | 30760888 |
| TH | tyrosine hydroxylase | 7054 | ENSG00000180176 | 21176768 |
| TK1 | thymidine kinase 1 | 7083 | ENSG00000167900 | 19468287 |
| TK2 | thymidine kinase 2 | 7084 | ENSG00000166548 | 32638267 |
| TPH1 | tryptophan hydroxylase 1 | 7166 | ENSG00000129167 | 19468287 |
| TPH2 | tryptophan hydroxylase 2 | 121278 | ENSG00000139287 | 19468287 |
| TYMP | thymidine phosphorylase | 1890 | ENSG00000025708 | 19468287 |
| TYR | tyrosinase | 7299 | ENSG00000077498 | 19468287 |
| UCK1 | uridine-cytidine kinase 1 | 83549 | ENSG00000130717 | 19468287 |
| UCK2 | uridine-cytidine kinase 2 | 7371 | ENSG00000143179 | 19468287 |
| UCKL1 | uridine-cytidine kinase 1 like 1 | 54963 | ENSG00000198276 | 19468287 |
| UGDH | UDP-glucose 6-dehydrogenase | 7358 | ENSG00000109814 | 19468287 |
| UGT2B4 | UDP glucuronosyltransferase family 2 member B4 | 7363 | ENSG00000156096 | 19468287 |
| XDH | xanthine dehydrogenase | 7498 | ENSG00000158125 | 19468287 |

**Table.S3 List of CPTAC cancer types.**

| **Project (Abbreviations)** | **Cancer Type** | **Primary Site** | **Tumor** | **Normal** |
| --- | --- | --- | --- | --- |
|  |  |  |  |  |
|  |  |  |  |  |
| **CRC** | Colorectal Cancer | Colorectal | 95 | 100 |
| **BRCA** | Breast Invasive Carcinoma | Breast | 133 | 18 |
| **KIRC** | Kidney Renal Clear Cell Carcinoma | Kidney | 110 | 84 |
| **LUAD** | Lung Adenocarcinoma | Lung | 113 | 102 |
| **UCEC** | Uterine Corpus Endometrial Carcinoma | Uterus | 100 | 31 |

**Table.S4 Dysregulation levels of metabolic rate-limiting enzymes in CRC.**

| **SYMBOL** | **ENSEMBLID** | **FC** | **P** |
| --- | --- | --- | --- |
| ACACA | ENSG00000278540 | 1.80575 | 2.90E-16 |
| ACACB | ENSG00000076555 | 0.31046 | 8.26E-30 |
| ACADL | ENSG00000115361 | 0.32243 | 1.05E-18 |
| ACADS | ENSG00000122971 | 0.25307 | 5.05E-29 |
| ACO1 | ENSG00000122729 | 0.92137 | 0.005656 |
| ACO2 | ENSG00000100412 | 0.52572 | 2.87E-25 |
| ACOX1 | ENSG00000161533 | 0.39187 | 2.22E-19 |
| ACOX2 | ENSG00000168306 | 1.1107 | 0.725599 |
| ACOX3 | ENSG00000087008 | 0.85902 | 9.66E-05 |
| ACSL1 | ENSG00000151726 | 1.23352 | 0.02076 |
| ADH1A | ENSG00000187758 | 0.17454 | 2.03E-29 |
| ADH7 | ENSG00000196344 | 0.58271 | 0.487454 |
| ADK | ENSG00000156110 | 1.9793 | 9.33E-19 |
| AKR1A1 | ENSG00000117448 | 1.18537 | 0.000891 |
| AKR1B1 | ENSG00000085662 | 0.70653 | 1.27E-09 |
| ALAD | ENSG00000148218 | 0.65666 | 8.34E-21 |
| ALAS1 | ENSG00000023330 | 0.74708 | 4.31E-10 |
| ALAS2 | ENSG00000158578 | 0.43017 | 5.36E-07 |
| ALDH1A1 | ENSG00000165092 | 0.5322 | 1.32E-14 |
| ALDH1A2 | ENSG00000128918 | 1.82509 | 2.88E-05 |
| ALDH1A3 | ENSG00000184254 | 1.43936 | 0.115399 |
| ALDH1B1 | ENSG00000137124 | 1.67843 | 1.38E-09 |
| ALDH2 | ENSG00000111275 | 0.80983 | 1.08E-07 |
| ALDH3A1 | ENSG00000108602 | 1.11226 | 0.023095 |
| ALDH3A2 | ENSG00000072210 | 0.6939 | 3.63E-14 |
| ALDH7A1 | ENSG00000164904 | 1.08817 | 0.413518 |
| ALDH9A1 | ENSG00000143149 | 0.83024 | 4.79E-09 |
| ALOX5 | ENSG00000012779 | 1.39894 | 0.981173 |
| AMD1 | ENSG00000123505 | 1.12559 | 0.010257 |
| APRT | ENSG00000198931 | 1.6784 | 6.92E-17 |
| ASS1 | ENSG00000130707 | 0.91016 | 0.941771 |
| BACE1 | ENSG00000186318 | 1.14976 | 0.138877 |
| BDH1 | ENSG00000161267 | 0.64681 | 9.44E-13 |
| BDH2 | ENSG00000164039 | 0.57274 | 6.05E-15 |
| CHAT | ENSG00000070748 | 0.05396 | 1.96E-40 |
| CHKB | ENSG00000100288 | 1.6605 | 1.05E-09 |
| COX4I1 | ENSG00000131143 | 0.88601 | 0.00054 |
| COX5B | ENSG00000135940 | 1.01533 | 0.307731 |
| COX6A1 | ENSG00000111775 | 0.79534 | 2.42E-07 |
| COX6A2 | ENSG00000156885 | 0.5586 | 1.36E-13 |
| COX6B1 | ENSG00000126267 | 0.96568 | 0.052565 |
| COX6C | ENSG00000164919 | 0.93716 | 0.000643 |
| CPS1 | ENSG00000021826 | 1.6733 | 0.389557 |
| CPT1A | ENSG00000110090 | 0.4809 | 1.54E-22 |
| CPT1B | ENSG00000205560 | 1.84262 | 1.89E-05 |
| CPT2 | ENSG00000157184 | 0.40993 | 1.34E-28 |
| CS | ENSG00000062485 | 0.92121 | 0.002582 |
| CTPS1 | ENSG00000171793 | 2.39444 | 2.82E-24 |
| CYP11B2 | ENSG00000179142 | 15.1022 | 0.32494 |
| CYP7A1 | ENSG00000167910 | 1.52518 | 1.93E-06 |
| DCK | ENSG00000156136 | 1.03574 | 0.446787 |
| DDC | ENSG00000132437 | 0.92889 | 0.247541 |
| DECR1 | ENSG00000104325 | 0.70159 | 2.90E-13 |
| DGAT2 | ENSG00000062282 | 4.1212 | 2.08E-25 |
| DHCR24 | ENSG00000116133 | 0.97176 | 0.687736 |
| DHCR7 | ENSG00000172893 | 2.77607 | 1.41E-23 |
| DHODH | ENSG00000102967 | 1.73509 | 4.73E-23 |
| DHPS | ENSG00000095059 | 1.40793 | 2.17E-14 |
| DLD | ENSG00000091140 | 0.80636 | 1.36E-09 |
| DLST | ENSG00000119689 | 0.76619 | 2.98E-14 |
| DPYD | ENSG00000188641 | 0.32664 | 2.02E-24 |
| DTYMK | ENSG00000168393 | 2.26739 | 2.65E-24 |
| F2 | ENSG00000180210 | 15.3662 | 1.57E-14 |
| FADS1 | ENSG00000149485 | 2.99445 | 3.92E-12 |
| FADS2 | ENSG00000134824 | 3.76392 | 3.12E-08 |
| FBP1 | ENSG00000165140 | 0.90717 | 0.27984 |
| FBP2 | ENSG00000130957 | 1.13145 | 0.000147 |
| G6PC1 | ENSG00000131482 | 0.0197 | 7.48E-17 |
| G6PC2 | ENSG00000152254 | 0.5208 | 1.78E-08 |
| G6PD | ENSG00000160211 | 1.78069 | 5.95E-19 |
| GAD1 | ENSG00000128683 | 42.0951 | 1.81E-27 |
| GAD2 | ENSG00000136750 | 1.6975 | 3.05E-06 |
| GCH1 | ENSG00000131979 | 0.90946 | 0.004878 |
| GFPT1 | ENSG00000198380 | 1.00458 | 0.304897 |
| GFPT2 | ENSG00000131459 | 1.05372 | 0.016097 |
| GGT1 | ENSG00000100031 | 0.78668 | 0.004993 |
| GLO1 | ENSG00000124767 | 1.81196 | 2.01E-16 |
| GNE | ENSG00000159921 | 0.55511 | 2.23E-13 |
| GPAM | ENSG00000119927 | 1.01033 | 0.118902 |
| GPD2 | ENSG00000115159 | 0.70172 | 5.05E-13 |
| H6PD | ENSG00000049239 | 0.94011 | 0.065609 |
| HDC | ENSG00000140287 | 0.41167 | 1.39E-15 |
| HK1 | ENSG00000156515 | 0.92866 | 0.061234 |
| HK2 | ENSG00000159399 | 0.4619 | 2.67E-14 |
| HK3 | ENSG00000160883 | 1.51575 | 0.464892 |
| HMGCR | ENSG00000113161 | 0.79773 | 0.000418 |
| HMGCS1 | ENSG00000112972 | 1.05328 | 0.081737 |
| HMGCS2 | ENSG00000134240 | 0.23388 | 1.03E-21 |
| HMOX1 | ENSG00000100292 | 0.37639 | 1.21E-21 |
| HMOX2 | ENSG00000103415 | 1.3147 | 6.77E-11 |
| HSD17B6 | ENSG00000025423 | 0.37092 | 5.65E-14 |
| HSD3B1 | ENSG00000203857 | 0.47049 | 0.039773 |
| HSD3B2 | ENSG00000203859 | 0.02123 | 3.54E-25 |
| IDH1 | ENSG00000138413 | 0.92259 | 0.013384 |
| IDH2 | ENSG00000182054 | 1.46059 | 2.10E-06 |
| IDO1 | ENSG00000131203 | 1.74278 | 0.17834 |
| IDO2 | ENSG00000188676 | 0.86082 | 0.000865 |
| IMPDH1 | ENSG00000106348 | 3.37967 | 1.49E-28 |
| IMPDH2 | ENSG00000178035 | 1.99876 | 2.21E-25 |
| KHK | ENSG00000138030 | 1.13735 | 3.10E-11 |
| KMO | ENSG00000117009 | 0.65249 | 3.20E-08 |
| LDHA | ENSG00000134333 | 1.56942 | 9.27E-14 |
| LIPE | ENSG00000079435 | 1.37353 | 2.99E-07 |
| LPCAT2 | ENSG00000087253 | 1.92135 | 1.20E-11 |
| LTA4H | ENSG00000111144 | 1.31052 | 4.74E-09 |
| LTC4S | ENSG00000213316 | 0.58739 | 5.88E-06 |
| MYLK | ENSG00000065534 | 0.10412 | 7.79E-26 |
| MYLK2 | ENSG00000101306 | 1.93616 | 2.21E-09 |
| MYLK3 | ENSG00000140795 | 0.64568 | 4.10E-07 |
| NAMPT | ENSG00000105835 | 1.40756 | 4.47E-05 |
| NAT1 | ENSG00000171428 | 0.40267 | 1.53E-22 |
| NAT2 | ENSG00000156006 | 0.33878 | 4.73E-23 |
| OGDH | ENSG00000105953 | 0.72468 | 1.74E-14 |
| OGDHL | ENSG00000197444 | 0.9015 | 1.26E-08 |
| OXCT1 | ENSG00000083720 | 0.78566 | 1.43E-05 |
| PAH | ENSG00000171759 | 53.1175 | 2.31E-17 |
| PCK1 | ENSG00000124253 | 0.16024 | 1.79E-24 |
| PCK2 | ENSG00000100889 | 0.65842 | 1.30E-09 |
| PCYT1A | ENSG00000161217 | 0.80444 | 4.31E-13 |
| PCYT1B | ENSG00000102230 | 0.56754 | 1.16E-09 |
| PEPD | ENSG00000124299 | 0.95618 | 0.349228 |
| PFKM | ENSG00000152556 | 1.50754 | 5.32E-11 |
| PFKP | ENSG00000067057 | 1.23011 | 0.026972 |
| PHGDH | ENSG00000092621 | 3.53254 | 5.60E-11 |
| PIK3C3 | ENSG00000078142 | 0.74786 | 2.65E-15 |
| PKLR | ENSG00000143627 | 1.36969 | 0.237249 |
| PKM | ENSG00000067225 | 2.03349 | 1.59E-22 |
| PLAT | ENSG00000104368 | 0.69577 | 1.41E-10 |
| PLAU | ENSG00000122861 | 4.54035 | 7.79E-26 |
| PNPLA2 | ENSG00000177666 | 0.92963 | 0.011892 |
| PTGR1 | ENSG00000106853 | 0.54042 | 2.08E-12 |
| PTGS1 | ENSG00000095303 | 0.14825 | 3.14E-28 |
| PTGS2 | ENSG00000073756 | 1.5123 | 0.327313 |
| PYGB | ENSG00000100994 | 1.24831 | 0.553704 |
| PYGL | ENSG00000100504 | 0.93966 | 0.006516 |
| PYGM | ENSG00000068976 | 0.07398 | 1.52E-27 |
| QPRT | ENSG00000103485 | 2.32032 | 4.46E-07 |
| RDH5 | ENSG00000135437 | 0.23018 | 1.17E-31 |
| REN | ENSG00000143839 | 5.90688 | 0.00014 |
| RRM1 | ENSG00000167325 | 1.45788 | 3.26E-12 |
| RRM2 | ENSG00000171848 | 2.82072 | 7.97E-24 |
| RRM2B | ENSG00000048392 | 0.98237 | 0.06943 |
| SAT1 | ENSG00000130066 | 1.32131 | 0.001874 |
| SAT2 | ENSG00000141504 | 0.7714 | 4.14E-06 |
| SCD | ENSG00000099194 | 5.36754 | 3.46E-26 |
| SCD5 | ENSG00000145284 | 0.90912 | 0.061859 |
| SLC6A4 | ENSG00000108576 | 0.75343 | 0.211028 |
| SOAT1 | ENSG00000057252 | 0.769 | 7.44E-08 |
| SOAT2 | ENSG00000167780 | 0.54201 | 0.79793 |
| SPTLC1 | ENSG00000090054 | 1.00945 | 0.877055 |
| SPTLC2 | ENSG00000100596 | 0.8926 | 0.002948 |
| SQLE | ENSG00000104549 | 3.23416 | 2.66E-21 |
| STS | ENSG00000101846 | 0.69704 | 2.70E-12 |
| TAT | ENSG00000198650 | 0.08138 | 2.47E-28 |
| TDO2 | ENSG00000151790 | 4.41131 | 1.39E-08 |
| TH | ENSG00000180176 | 12.1486 | 5.32E-18 |
| TK1 | ENSG00000167900 | 2.04484 | 5.06E-15 |
| TK2 | ENSG00000166548 | 1.06118 | 0.475948 |
| TPH1 | ENSG00000129167 | 0.10918 | 3.54E-29 |
| TPH2 | ENSG00000139287 | 0.91864 | 8.33E-07 |
| TYMP | ENSG00000025708 | 1.55922 | 0.004138 |
| TYR | ENSG00000077498 | 18.4028 | 0.026145 |
| UCK1 | ENSG00000130717 | 0.91938 | 0.001328 |
| UCK2 | ENSG00000143179 | 1.18058 | 0.000302 |
| UCKL1 | ENSG00000198276 | 2.70825 | 9.69E-27 |
| UGDH | ENSG00000109814 | 0.31276 | 1.29E-28 |
| UGT2B4 | ENSG00000156096 | 24.1687 | 0.255357 |
| XDH | ENSG00000158125 | 0.21791 | 3.89E-25 |

**Table.S5 List of antibodies used in this study.**

| Protein | Company | Catalog No. | Source | Dilution |
| --- | --- | --- | --- | --- |
| IMPDH1 | Abclonal | A9497 | Rabbit polyclonal antibody | 1:1000 (WB) |
| IMPDH2 | Abclonal | A9208 | Rabbit monoclonal antibody | 1:1000 (WB) |
| GAPDH | Abbkine | ABP50163 | Rabbit polyclonal antibody | 1:2000 (WB) |
| c-Myc | Santa Cruz | Sc-40 | Mouse monoclonal antibody | 1:200 (WB) |
| c-Myc | Abclonal | A1309 | Rabbit polyclonal antibody | 1:1000 (WB) |
| HA | Sigma | H6908 | Rabbit monoclonal antibody | 1:5000 (WB) |
| HA | Abclonal | AE008 | Mouse monoclonal antibody | 1:2000 (WB) |
| Flag | Proteintech | 20543-1-AP | Rabbit polyclonal antibody | 1:3000 (WB) |

**Table.S6 List of primer and shRNA sequences used in this study.**

**Primers for construction**

| Insert | Forward Primer (5’>3’) | Reverse Primer (5’>3’) |
| --- | --- | --- |
| Flag-IMPDH1 | CGCGCGGCCGCATGGAGGGGCCACTCACTCCACC | CGCCTCGAGGTACAGCCGCTTTTCGTAAGAG |
| Flag- IMPDH1-△CBS | ACGAGGTGCGGAAGGTCTGTGGGGCAGCTGTGGGCA | TGCCCACAGCTGCCCCACAGACCTTCCGCACCTCGT |
| Flag- IMPDH1-C | CGCGCGGCCGCATGTGTGGGGCAGCTGTGGGCAC | CGCCTCGAGGTACAGCCGCTTTTCGTAAGAG |
| HA-IMPDH2 | CGCGTCGACCATGGCCGACTACCTGATTAGTG | CGCGCGGCCGCTCAGAAAAGCCGCTTCTCATAC |
| HA-IMPDH2-△CBS | CAATGAAGTTCGGAAAGTGTGTGGGGCAGCCATTGGCA | TGCCAATGGCTGCCCCACACACTTTCCGAACTTCATTG |
| HA-IMPDH2-C | CGCGTCGACCATGTGTGGGGCAGCCATTGGC | CGCGCGGCCGCTCAGAAAAGCCGCTTCTCATAC |
| Flag-Myc | CGCGCGGCCGCATGCCCCTCAACGTTAGC | CGCGTCGACCGCACAAGAGTTCCGTAGCT |

**qPCR primers**

| Amplicons | Forward Primer (5’>3’) | Reverse Primer (5’>3’) |
| --- | --- | --- |
| *IMPDH1* | CAGCAGGTGTGACGTTGAAAG | AGCTCATCGCAATCATTGACG |
| *IMPDH2* | GCGCTTACAGGCGGTATTG | AAAACATCCCGCACGCGAT |
| *MYLK* | CCCGAGGTTGTCTGGTTCAAA | GCAGGTGTACTTGGCATCGT |
| *XDH* | CCAAATTGCTGCATGAACCAG | TGCTTCCGAGGAGTGTCTTTC |
| *DPYD* | GGCGGACATCGAGAGTATCCT | TTCTTGGCCGAAGTGGAACAC |
| *UGDH* | TGCCCAGAGAATAAGCAGCAT | CCATTCCAATCGCTGTTGCTA |
| *PTGS1* | CGCCAGTGAATCCCTGTTGTT | AAGGTGGCATTGACAAACTCC |
| *GAPDH* | GGAGCGAGATCCCTCCAAAAT | GGCTGTTGTCATACTTCTCATGG |

**shRNA sequences**

| shRNA | Sequences (5’>3’) |
| --- | --- |
| shIMPDH1-1# | GGAAGCTGCCTATCGTCAATG |
| shIMPDH1-2# | GCACCCGTGAGGATGACAAAT |
| shMyc-1# | CCTGAGACAGATCAGCAACAA |
| shMyc-2# | CAGTTGAAACACAAACTTGAA |
| shIMPDH2-1# | GGACAGACCTGAAGAAGAA |
| shIMPDH2-2# | GCAGTGTACAAGGTGTCAGAG |
| shCtrl | TTCTCCGAACGTGTCACGT |

**Primers for ChIP**

| Amplicons | Forward Primer (5’>3’) | Reverse Primer (5’>3’) |
| --- | --- | --- |
| *IMPDH1-RE* | ACTGCTGCAGGCCGGCTAC | GTCTGCATCCCCCAACCAAAG |
| *IMPDH2-RE* | CTATACGCATGCGCTGTTTC | TGCCCCCACTAATCGGTAG |
| *GAPDH* | GGAGCGAGATCCCTCCAAAAT | GGCTGTTGTCATACTTCTCATGG |
